# Oncodriver inhibition and CD4^+^ Th1 cytokines cooperate through Stat1 activation to induce tumor senescence and apoptosis in HER2+ and triple negative breast cancer: implications for combining immune and targeted therapies

**DOI:** 10.18632/oncotarget.25208

**Published:** 2018-05-01

**Authors:** Cinthia Rosemblit, Jashodeep Datta, Lea Lowenfeld, Shuwen Xu, Amrita Basu, Krithika Kodumudi, Doris Wiener, Brian J. Czerniecki

**Affiliations:** ^1^ Harrison Department of Surgical Research, Department of Surgery, University Pennsylvania Perelman School of Medicine, Philadelphia, PA, USA; ^2^ Department of Clinical Science, H Lee Moffitt Cancer Center, Tampa, FL, USA

**Keywords:** CD4^+^ T-helper immunity, HER2/neu, triple negative, breast cancer

## Abstract

In patients with HER2-expressing breast cancer many develop resistance to HER2 targeted therapies. We show that high and intermediate HER2-expressing cancer cell lines are driven toward apoptosis and tumor senescence when treated with either CD4^+^ Th1 cells, or Th1 cytokines TNF-α and IFN-γ, in a dose dependent manner. Depletion of HER2 activity by either siRNA or trastuzumab and pertuzumab, and subsequent treatment with either anti-HER2 Th1 cells or TNF-α and IFN-γ resulted in synergistic increased tumor senescence and apoptosis in cells both sensitive and cells resistant to trastuzumab which was inhibited by neutralizing anti-TNF-α and IFN-γ. Th1 cytokines induced minimal senescence or apoptosis in triple negative breast cancer cells (TNBC); however, inhibition of EGFR in combination with Th1 cytokines sensitized those cells causing both senescence and apoptosis. TNF-α and IFN-γ led to increased Stat1 phosphorylation through serine and tyrosine sites and a compensatory reduction in Stat3 activation. Single agent IFN-γ enhanced Stat1 phosphorylation on tyrosine 701 and similar effects were observed in combination with TNF-α and EGFR inhibition. These results demonstrate Th1 cytokines and anti-oncodriver blockade cooperate in causing tumor senescence and apoptosis in TNBC and HER2-expressing breast cancer, suggesting these combinations could be explored as non-cross-reactive therapy preventing recurrence in breast cancer.

## INTRODUCTION

Breast cancer is the most common malignancy in women worldwide. More than 240,000 patients will be diagnosed with breast cancer and more than 40,000 will die in 2017 of this disease in the United States [1–3].

The receptor tyrosine kinase human epidermal growth factor receptor 2 (HER2) is overexpressed in 25% of breast cancers and is associated with a poor prognosis [4–6]. HER2 belongs to the family of type I receptor tyrosine kinases, which includes three other homologous proteins: HER1 (EGFR/ErbB1), HER3 (ErbB3), and HER4 (ErbB4). These proteins are essential for amplification, and overexpression correlates with enhanced tumor aggressiveness in breast cancer and other malignancies [7]. HER2 has been widely implicated in malignant transformation, cell survival, motility and invasion in breast cancers. The heterodimer HER2-HER3 has the strongest interaction, the most potent ligand-induced tyrosine phosphorylation and downstream signaling, and functions as an oncogenic unit; thus, HER3 acts as a critical partner for both EGFR and HER2. Given their oncogenic capacity and their frequently aberrant expression or deregulation in human tumors, members of the HER family are appealing targets for approved therapeutics and novel anticancer agents [8, 9]. Targeted therapies have dramatically improved outcomes; however, many patients develop resistance or recur with resistant tumors.

Triple negative breast cancer (TNBC) does not express HER2, ER and PR and constitutes 20% of all breast cancers with poor prognosis and high risk of relapse [10, 11]. Overexpression of EGFR is a common phenomenon in TNBC and nuclear EGFR expression correlates with a more aggressive clinical behavior in these cancers [12].

Signal transducers and activators of transcription (Stats) constitute a family of seven proteins that play a key role in immune response, cell growth, differentiation, antiviral activity and homeostasis by directing the transcriptional response of cytokines and growth factors [13]. Stats are latent transcription factors in the cytoplasm that become activated by tyrosine phosphorylation, dimerize and translocate to the nucleus where they bind to DNA. For maximal activation of transcription Stats require both tyrosine and serine phosphorylation [14, 15]. Accumulated evidence indicated that Stat1 mediates anti-proliferative effects by inducing upregulation of cell cycle inhibitors and apoptosis genes in several tissues and *Stat1*-null mice are more prone to tumor development than controls [15–19].

Our group has recently identified the possible immune underpinnings underlying such events; across a broad tumorigenic continuum, we established not only that the anti-HER2 CD4^+^ T-helper type 1 (Th1) immune response is progressively lost during disease progression [20], but also that an anti-HER2 Th1 immune deficit is associated with unfavorable clinicopathologic outcomes, i.e., disease recurrence and incomplete pathologic response following neoadjuvant therapy [21, 22]. To this end, there is a paucity of information on the impact of Th1 cytokines on HER2-expressing breast cancer cells [23].

Cellular senescence is an irreversible proliferation arrest that occurs in normal tissue following an excessive number of cell divisions or induced by stress. Oncogene addiction is a phenomenon that describes the dependency of tumor cells on a single activated oncogenic protein or pathway to maintain their malignant properties [24]. Thus, oncogene-induced senescence is an antitumor barrier that prevents the expansion of early neoplastic cells before they become malignant [25, 26]. The senescent cells remain metabolically active and release factors collectively termed ‘senescence-associated secretory phenotype’ or ‘senescence messaging secretome’ [27–29]. The senescence secretome includes the components necessary to establish and maintain senescence and proinflammatory cytokines that attract cellular components of the innate and adaptive immune response that mediate clearance of senescent cells [29–32].

In this study we sought to (1) determine whether Th1 cytokines, tumor necrosis factor alpha (TNF-α) and interferon gamma (IFN-γ), induce senescence and apoptosis in HER2-expressing breast cancer cells, assess the impact of (2) Th1 cytokines combined with simultaneous HER2 and HER3 blockade in HER2-expressing cells and (3) EGFR and HER3 blockade in TNBC cells. These results reveal a paradigm in which synergism between Th1 cytokines and a multivalent, targeted therapy can be explored to effectively eliminate residual breast cancer cells by inducing tumor senescence and apoptosis to targeted therapy and prevent recurrence.

## RESULTS

### Th1 cytokines TNF-α and IFN-γ synergize to induce senescence in breast cancer cells

We first tested the ability of Th1 cytokines to induce a specific senescence response in tumor cells. All the cell lines used here are color coded as identifier throughout the figures as shown in Table 1: SK-BR-3 (green), BT-474 (beige), MCF-7 (grey), T-47D (blue), HCC-1419 (orange), MDA-MB-231 (pink), MDA-MB-468 (black), Hs-578 (red) and HCC-1143 (purple) and JIMT-1 (yellow).

**Table 1:** Breast cancer cell lines listed by their HER2 expression levels

Breast cancer phenotype	Cell line
HER2	SK-BR-3
	BT-474
	JIMT-1
	HCC-1419
ER^pos^ intermediate HER2	T-47D
	MCF-7
Triple negative EGFR+	HCC-1143
	MDA-MB-468
	Hs-578
	MDA-MB-231

Schematic representation of breast cancer cell lines by their HER2 expression levels.

**Figure 1:**
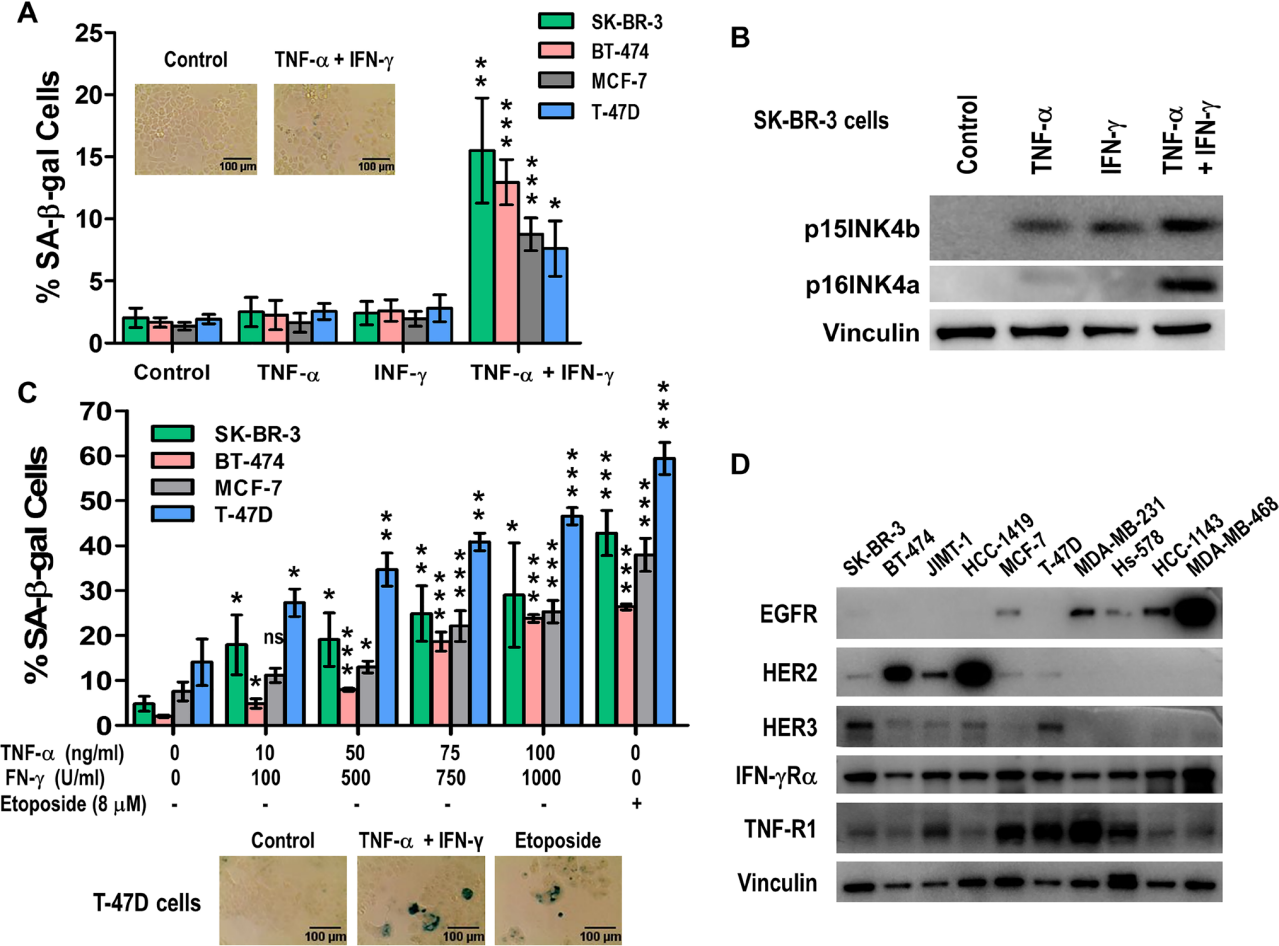
Th1 cytokines, TNF-α and IFN-γ, synergize to induce senescence in breast cancer cells in a dose dependent manner (**A**) SK-BR-3, BT-474, MCF-7 and T-47D) breast cancer cell lines untreated or incubated with 10 ng/ml TNF-α alone, 100 U/ml IFN-γ alone, or TNF-α and IFN-γ in combination. Only paired cytokines induced senescence. Densitometric analysis presented as % of SA-β-gal-positive cells, mean ± SD (*n* = 3), ^*^p < 0.05, ^**^p < 0.01, ^***^p < 0.001. *Inset:* representative data from 1 of 3 independent experiments on SK-BR-3 cells. (**B**) p15INKb and p16INK4a expression of cells described in A were analyzed by western blot for SK-BR-3 cells. Vinculin was used as loading control. (**C**) SK-BR-3, BT-474, MCF-7 and T-47D breast cancer cells were untreated, treated with etoposide, or incubated with increasing concentrations of TNF-α and IFN-γ. *Top panel:* densitometric analysis presented as % of SA-β-gal-positive cells, mean ± SD (*n* = 3), ^*^p < 0.05, ^**^p < 0.01, ^***^p < 0.001. *Bottom panel*: representative data from 1 of 3 independent experiments on T-47D cells. (**D**) EGFR, HER2, HER3, IFNGR and TNFR expression in breast cancer cell lines as determined by western blot. Vinculin was used as loading control. Similar results were observed in 3 independent experiments.

SK-BR-3, BT-474, MCF-7 and T-47D were incubated with human recombinant TNF-α and IFN-γ alone or in designated combinations and subjected to senescence studies. The combination of both TNF-α and IFN-γ resulted in senescence induction of all cell lines, evidenced by increased senescence associated acidic β-galactosidase (SA-β-gal) staining (Figure 1A). Higher expression of the senescence-associated markers p15INK4b/CDKN2B and p16INK4a/CDKN2A were found in SK-BR-3 cells treated with combined cytokines (Figure 1B) compared with untreated cells or cells treated with each cytokine alone detected by western blot. Treating SK-BR-3, BT-474, MCF-7 and T-47D cells with increasing concentrations of TNF-α, 10 to 100 ng/ml, and IFN-γ , 100 to 1000 U/ml, demonstrated that induction of the senescent phenotype was dose dependent (Figure 1C).

### TNF-α and IFN-γ cytokines receptors are expressed in similar levels in breast cell lines

TNF-α and IFN-γ have each been shown to be active and critical at different phases of normal breast development. TNF-α is involved in proliferation, development, and branching morphogenesis of the normal mammary gland [33]. The receptor TNFR1 mediates TNF-α-induced proliferation of mammary epithelial cells, and the receptor TNFR2 induces casein accumulation [34]. Similarly, the active form of IFN-γ interacts with its receptor expressed on the surface of almost all normal cells [35, 36]. All the cell lines tested demonstrated similar TNF-α and IFN-γ receptors expression by western blot analysis (Figure 1D). The expression level of these two cytokine receptors is independent of the EGFR, HER2 or HER3 expression levels (Figure 1D).

### Dual blockade of HER2 and HER3 enhances Th1 cytokine-mediated senescence and apoptosis in breast cancer cells

**Figure 2:**
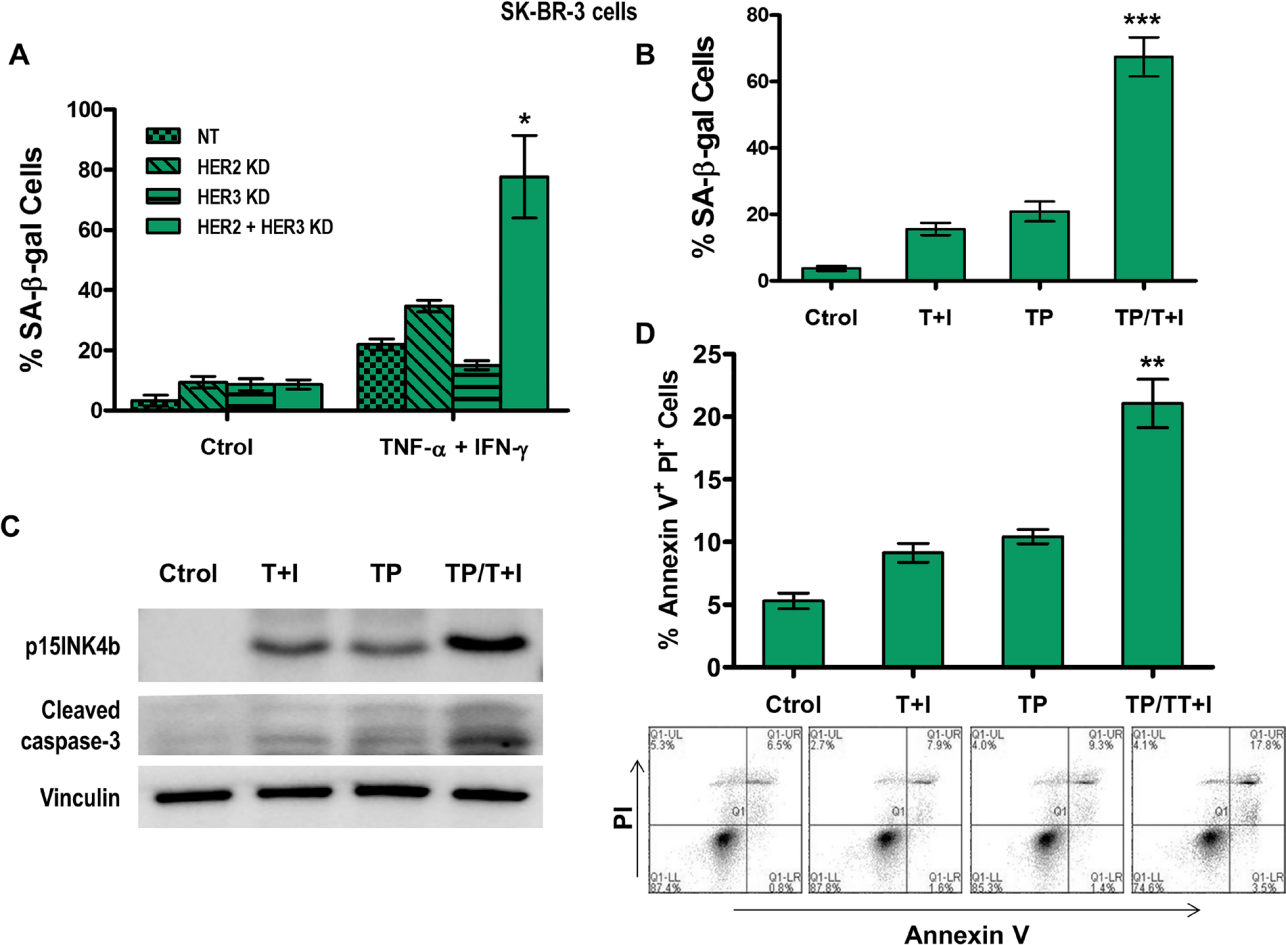
Combined HER2 and HER3 blockade enhances Th1 cytokine-mediated senescence and apoptosis in breast cancer cells (**A**) Densitometric analysis presented as % of SA-β-gal-positive SK-BR-3 cells transfected with non-target (NT), HER2, or HER3 siRNA, untreated or treated with 10 ng/ml TNF-α (Τ) and 100 U/ml IFN-γ (Ι), mean ± SD (*n* = 3), ^*^p < 0.005. (**B**) Densitometric analysis presented as % of SA-β-gal-positive SK-BR-3 cells untreated, treated with 10 ng/ml TNF-α and 100 U/ml IFN-γ (T+I), treated with 10 ug/ml of trastuzumab and pertuzumab (TP) or treated with the combination of both TNF-α and IFN-γ and trastuzumab and pertuzumab treatments (T+I/TP), mean ± SD (*n* = 3), ^***^p < 0.001 (**C**) p15INKb or cleaved caspase-3 expression of cells described in (B). Vinculin was used as loading control. Similar results were observed in 3 independent experiments. (**D**) Induction of apoptosis in SK-BR-3 cells was measured by staining for annexin V and PI expression in cells described in B, and analyzed by flow cytometry. *Top panel:* Densitometric analysis presented as % of annexin V^+^ PI^+^ cells, mean ± SEM (*n* = 3), ^**^p < 0.01. *Bottom panel*: Plots are representative data from 1 of 3 independent experiments. KD denotes knocked down.

The therapeutic benefit of blocking HER2/HER3 signaling in breast cancer has been demonstrated in both *in vitro* studies and clinically [9, 37]. We explored the senescent and apoptotic effects of Th1 cytokines in high and intermediate HER2-expressing cell lines blocked with HER2 and HER3 siRNA (Figure 2). Although the combined treatment of TNF-α and IFN-γ in HER3-knocked down SK-BR-3 cells did not significantly enhance the number of senescent cells, higher SA-β-gal staining was observed in cells treated with dual HER2/HER3-knocked down combined with Th1 cytokines (Figure 2A, *p* < 0.05). Similar results were found in MCF-7 cells (HER2^intermediate^, Supplementary Figure 1).

Trastuzumab, a humanized recombinant monoclonal antibody directed against the extracellular subdomain IV of HER2, inhibits ligand-independent dimerization, blocks downstream proliferation signaling pathways, and induces antibody-dependent cellular cytotoxicity (ADCC) [38, 39]. Pertuzumab, another humanized recombinant monoclonal antibody targeting the extracellular subdomain II of HER2, prevents ligand-dependent heterodimerization with other members of the HER family, which also inhibits proliferation signaling pathways and induces ADCC [40, 41]. Together both antibodies act in a complementary fashion. *In vitro*, the combination synergistically inhibited survival of HER2-overexpressing breast cancer cells and increased apoptosis [42]. In breast and non-small cell lung cancer xenograft models, the combination also enhanced the antitumor effect and induced tumor regression [43]. Most importantly, this combination improved progression-free and overall survival in metastatic breast cancer patients compared with trastuzumab alone [44].

We applied our paradigm of Th1 cytokine-induced senescence and apoptosis to a combination model, using TNF-α and IFN-γ treatment together with trastuzumab and pertuzumab. In HER2^high^ SK-BR-3 cells, we found that senescence increased synergistically in cells treated with the combination of cytokine and antibodies compared to untreated cells, cells treated with cytokines alone, or cells treated with antibodies alone, as measured by SA-β-gal staining (Figure 2B, *p* < 0.001) and p15INK4b expression (Figure 2C). Notably, the combined treatment not only induced a relatively higher percentage of blue senescent cells, but there were also significantly fewer cells overall. Increased apoptosis in an additive fashion was demonstrated by increased active caspase-3 expression (Figure 2C) and increased annexin V and propidium iodide positive cells (Figure 2D, *p* < 0.01).

### HER2-specific CD4^+^ Th1-mediated senescence and apoptosis in HER2-ovexpressing human breast cancer cells

**Figure 3:**
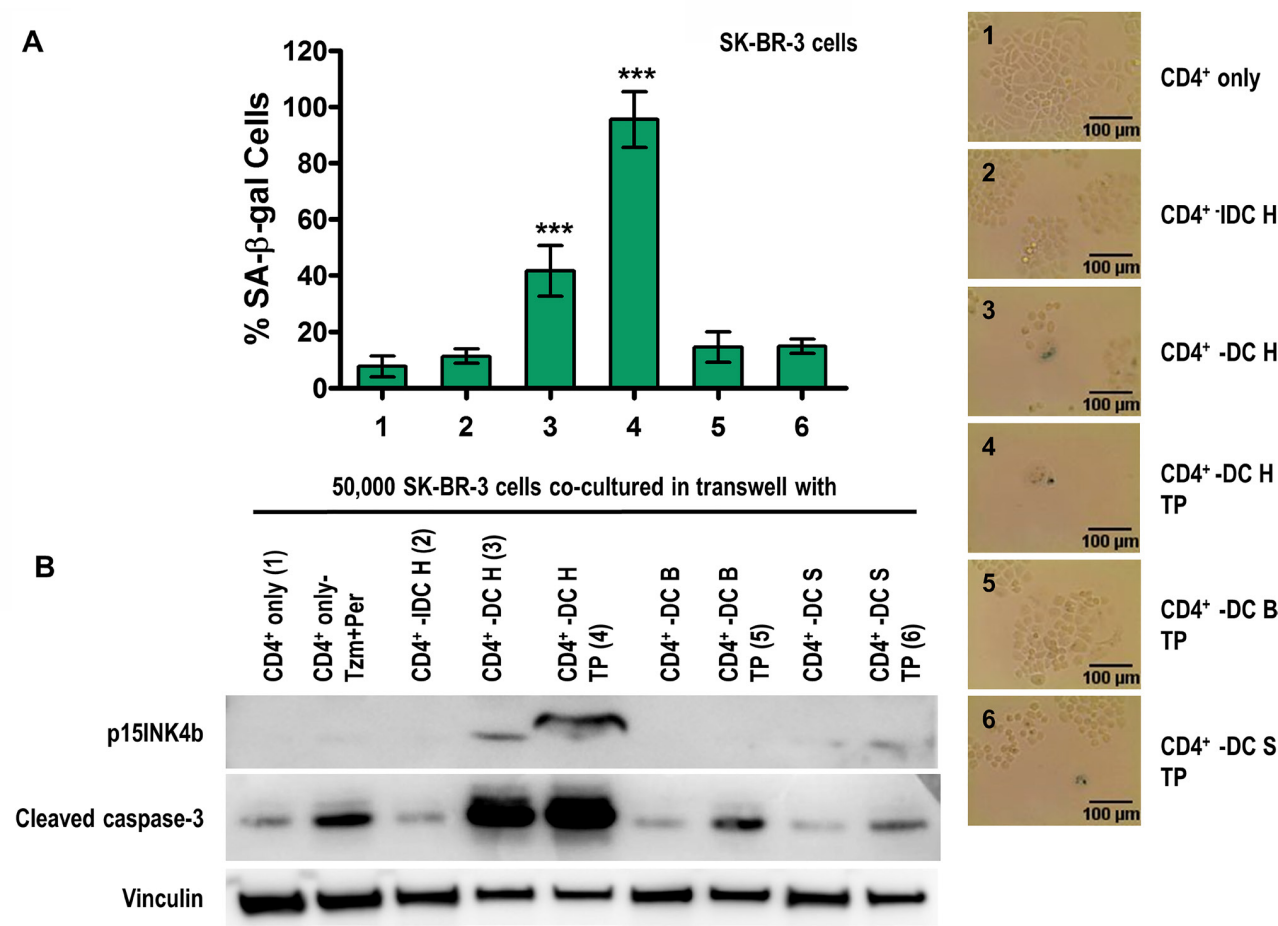
HER2-specific CD4^+^ Th1-mediated senescence and apoptosis of HER2-ovexpressing human breast cancer cells (**A**) SK-BR-3 cells co-cultured with CD4^+^ T-cells alone (CD4^+^ only (1)), CD4^+^ T-cells + HER2 peptide-pulsed immature dendritic cells (CD4^+^ IDC H (2)), CD4^+^ T-cells + HER2 peptide-pulsed mature dendritic cells (CD4^+^ DC H (3)), or CD4^+^ DC H with trastuzumab and pertuzumab (TP) (4), or CD4^+^ T-cells + irrelevant peptide-pulsed mature dendritic cells (BRAF (CD4^+^ DC B) (5); or survivin (CD4^+^ DC S)(6)), with TP. *Left panel:* densitometric analysis presented as % of SA-β-gal-positive cells, mean ± SD (*n* = 3), ^***^p < 0.001. *Right panel*: representative data from 1 of 3 independent experiments. (**B**) Increased p15INK4b and cleaved caspase-3 expression suggests induced senescence and apoptosis, respectively, when co-cultured with the DC H/CD4^+^ T-cells in presence of TP, but not from DC B, DC S and IDC H groups. Vinculin was used as loading control. Results are representative of 3 independent experiments.

**Figure 4:**
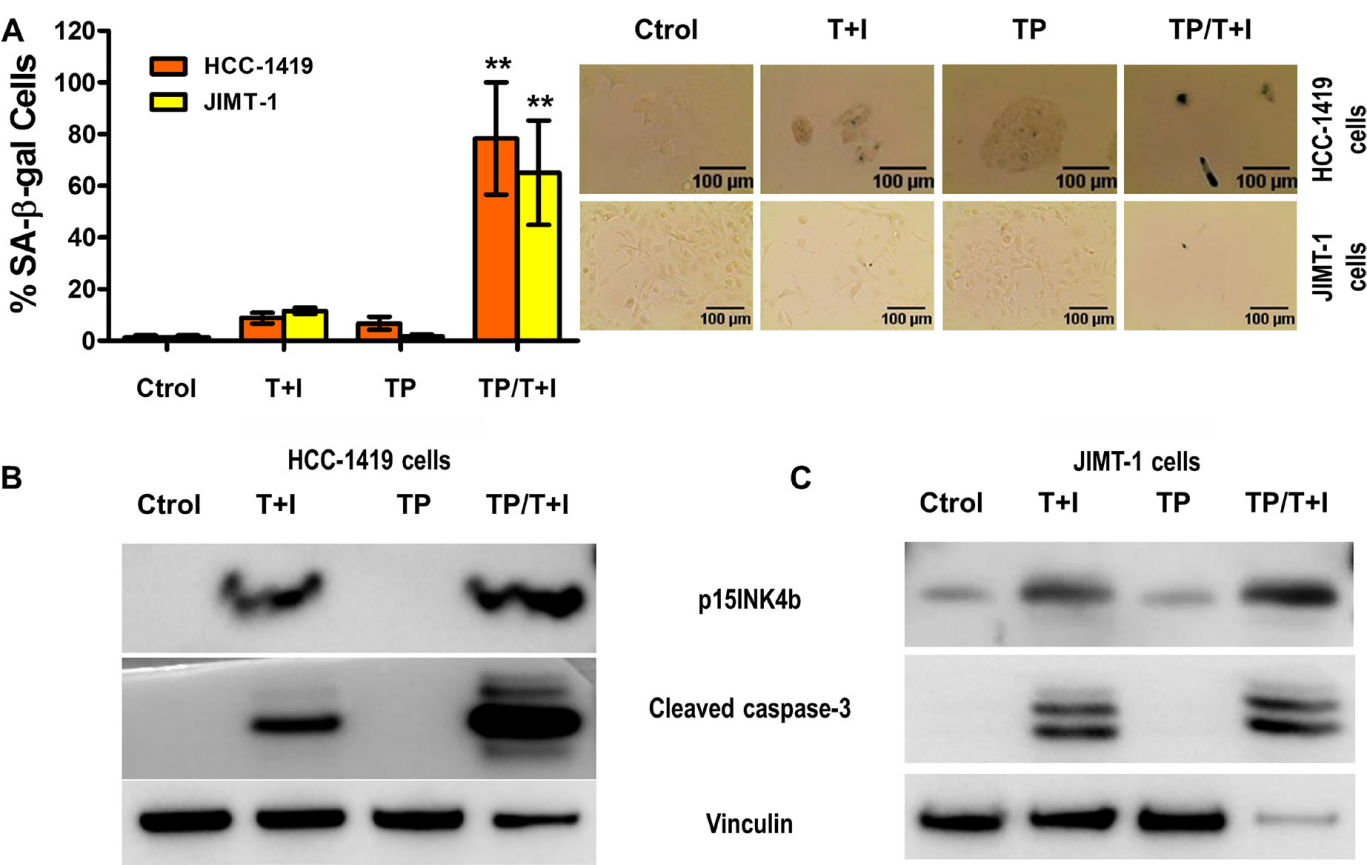
Th1 cytokines TNF-α and IFN-γ sensitize trastuzumab and pertuzumab resistant breast cancer cells to senescence and apoptosis induction (**A**) HCC-1419 and JIMT-1 cells, untreated, treated with 50 ng/ml TNF-α and 500 U/ml IFN-γ (T+I), or treated with 10 ug/ml of trastuzumab and pertuzumab (TP), or treated with both T+I/TP. *Left panel*, densitometric analysis presented as % of SA-β-gal-positive cells, mean ± SD (*n* = 3), ^**^p < 0.01. *Right panel*: representative data from 1 of 3 independent experiments in HCC-1419 cells (*top panel*) and JIMT-1 cells (*bottom panel*). (**B** and **C**) p15INKb and cleaved caspase-3 expression of HCC-1419 (B) and JIMT-1 (C) cells described in A. Vinculin was used as loading control. Similar results were observed in 3 independent experiments.

We confirmed our findings using Th1 cytokines produced by the CD4^+^ T-cells *ex vivo*. SK-BR-3 breast cancer cells co-cultured with CD4^+^ T-cells from breast cancer patients [45] primed with Class II HER2 peptides resulted in senescence and apoptosis of SK-BR-3 cells, evidenced by increased SA-β-gal staining (Figure 3A, 3B, *p* < 0.001) and p15INK4b and cleaved caspase-3 expression (Figure 3B, CD4^+^ - DC H, 3). CD4^+^ T-cells primed either with immature dendritic cells (CD4^+^ - IDC H (2)) or mature DCs plus irrelevant Class II peptides (BRAF: CD4^+^ - DC B (5); or survivin: CD4^+^ - DC S (6)) were not able to induce senescence or apoptosis of SK-BR-3 cells. Similar to the previously demonstrated synergistic effect, senescence and apoptosis were significantly augmented when trastuzumab and pertuzumab were added to the culture, evidenced by increased SA-β-gal staining (Figures 3A, 4, *p* < 0.001) and p15INK4b and cleaved caspase-3 expression (Figure 3B, CD4^+^ - DC H TP, 4).

We re-demonstrated this effect by co-culture of SK-BR-3 cells with the supernatant of CD4^+^ T-cells and DC combinations described above (data not shown). The Th1 cytokines, TNF-α and IFN-γ, obtained from co-culture of CD4^+^ T-cell and mature DC supernatants were confirmed using ELISA [20]. By both experimental approaches, senescence and apoptosis could be partially rescued by neutralizing TNF-α and IFN-γ with blocking antibodies (Supplementary Figures 2, 6). Also, by both experimental approaches, the effect was dose-dependent as increasing number of CD4^+^ and DC induced higher SA-β-gal staining and increased p15INK4b and cleaved caspase-3 expression (data not shown).

### Th1 cytokines TNF-α and IFN-γ sensitize trastuzumab and pertuzumab resistant breast cancer cells to senescence and apoptosis induction

We explored the potential for TNF-α and IFN-γ to induce senescence and apoptosis in trastuzumab and pertuzumab resistant cell lines. As shown in previous studies, we found that treatment with trastuzumab and pertuzumab blocked activation of AKT in sensitive cells T-47D, but did not prevent activation of AKT in two resistant cell lines HCC-1419 [46] and JIMT-1 [46, 47] (Supplementary Figure 3).

The treatment with TNF-α and IFN-γ induced senescence and apoptosis in a dose dependent manner in HCC-1419 and JIMT-1 (Supplementary Figure 4). As expected, when HCC-1419 and JIMT-1 cells were treated with trastuzumab and pertuzumab neither senescence nor apoptosis was elicited (Figure 4). However, the dual treatment with cytokines and targeted therapies induced significantly greater senescence as evidenced by SA-β-gal assay (Figure 4A, *p* < 0.01) and increased expression of p15INK4b in HCC-1419 cells (Figure 4B) and JIMT-1 cells (Figure 4C). Moreover, the combination of cytokines and antibodies also effectively induced cell death in HCC-1419 cells (Figure 4B) and JIMT-1 cells (Figure 4C). Th1 cytokines combined with HER2/HER3 blockade can cause tumor senescence and apoptosis even in cell lines resistant to trastuzumab and pertuzumab.

### HER2 and HER3 blockade enhances Th1-mediated Stat1 activation though Janus kinases and p38 MAPK in breast cancer cells

**Figure 5:**
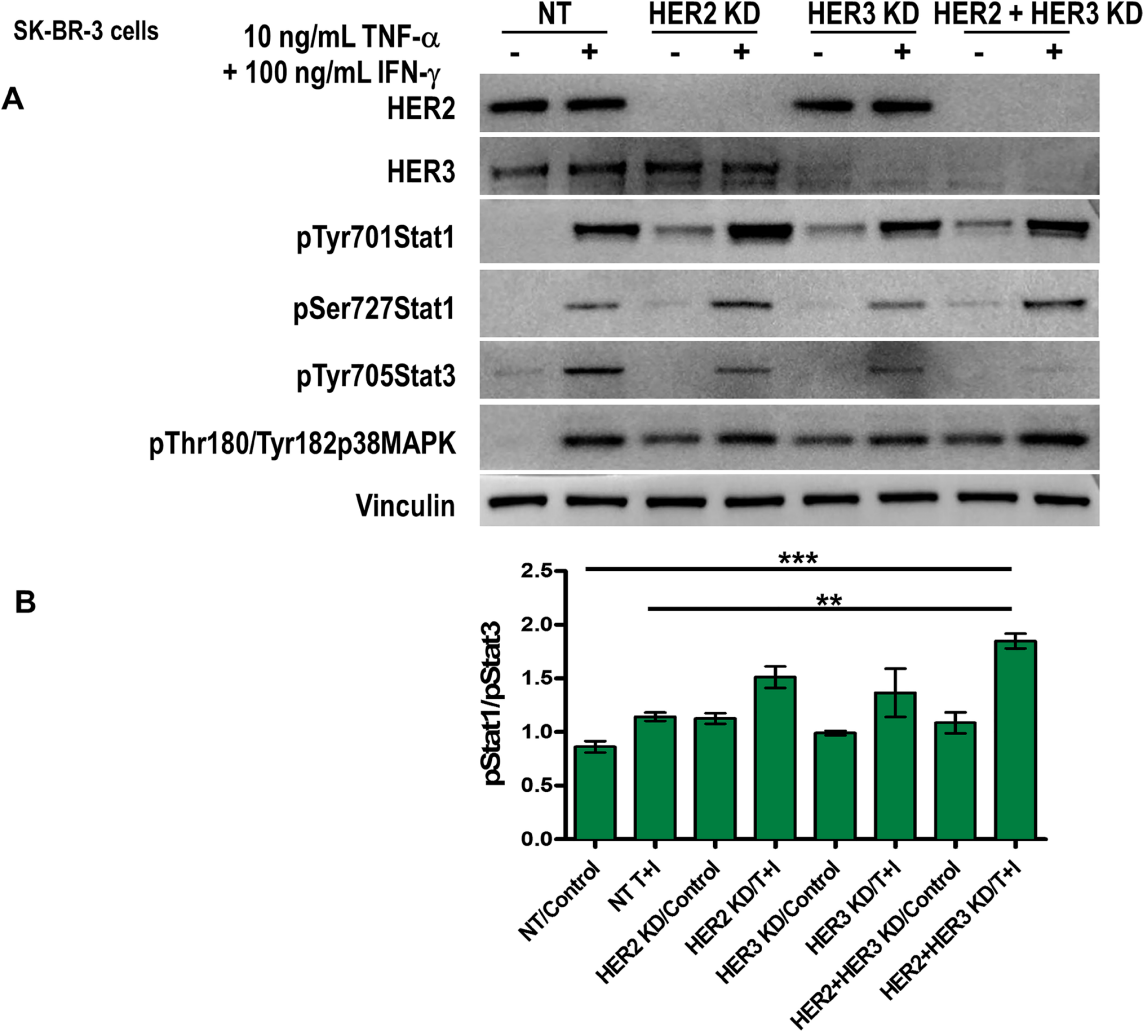
Th1 cytokines-mediated Stat1 activation increases in combination with HER2 and HER3 blockade (**A**) SK-BR-3 cells were transfected with non-target (NT), HER2, HER3 or HER2/HER3 siRNA, the cells were serum starved for 48 h and untreated or treated with 10 ng/ml TNF-α and 100 U/ml IFN-γ for 5 minutes. HER2 and HER3 expression, phospho-Stat1 tyrosine 701, phospho-Stat1 serine 727, phospho-Stat3 tyr 705, phospho-p38 MAPK threonine 180/ tyrosine 182 were determined by western blot. Vinculin was used as loading control. Similar results were observed in 3 independent experiments. (**B**) Densitometric analysis presented as mean of phospho-Stat1 tyrosine 701/phospho-Stat3 705 (arbitrary units, *n* = 3). KD denotes knocked down.

To unravel the mechanism that leads to senescence and apoptosis in breast cancer, we studied the activation of Stat1. We found that the treatment with TNF-α and IFN-γ for 5 min induced Stat1 on tyrosine 701 and on serine 727 phosphorylation residues by western blot in SK-BR-3 cells (Figure 5A). HER2 and HER3 knocked down by siRNA in combination with Th1 cytokines, TNF-α and IFN-γ further increased Stat1 phosphorylation (Figure 5A). Notably, HER2/HER3 blockade did not increase Th1 cytokines induced Stat1 tyrosine phosphorylation (Figure 5A). Similarly, treatment with TNF-α and IFN-γ induced p38 MAPK phosphorylation, which was also increased in HER2/HER3-depletd cells (Figure 5A). Conversely, HER2/HER3 blockade diminished Th1 cytokines induced tyrosine phosphorylation of another member of Stat family, Stat3 (Figure 5A). Constitutive activation of Stat3 has been detected at high frequency in diverse human cancer cell lines and tissues [48]. We compared relative level of phosphorylated Stat1 (Tyr701) and phosphorylated Stat3 (Tyr705) proteins and observed combination of HER2 and HER3 knock down with Th1 cytokines significantly increased ratio of phosphorylated Stat1:Stat3 (1:1.9) compared to untreated cells (*p =* 0.0001) and Th1 cytokine treatment alone (Figure 5B, pStat1:pStat3 = 1:1.2, *p =* 0.0042), indicating reversal of Stat phosphorylation and subsequent senescence and apoptosis effects on cancer cells. Similar results were obtained in T-47D cells (Supplemental Figure 5).

**Figure 6:**
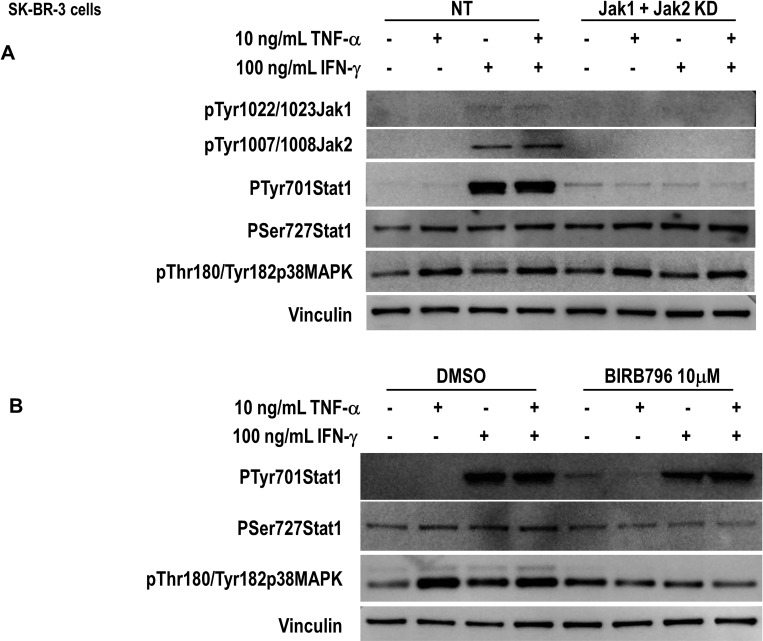
Th1-mediated Stat1 activation though Janus kinases and p38 MAPK in breast cancer cells (**A**) SK-BR-3 cells were transfected with non-target (NT), or a combination of JAK1 and JAk2 siRNA, untreated or treated with 10 ng/ml TNF-α, 100 U/ml IFN-γ or a combination of both for 5 minutes. Phospho-Jak1 tyrosines 1022/1023, phospho-Jak2 tyrosines 1007/1008, phospho-Stat1 tyrosine 701, phospho-Stat1 serine 727, and phospho-p38 MAPK threonine 180/tyrosine 182 were determined by western blot. Vinculin was used as loading control. Similar results were observed in 3 independent experiments. (**B**) SK-BR-3 cells were pretreated with BIRB796 10 µM for 90 min and untreated or treated with 10 ng/ml TNF-α, 100 U/ml IFN-γ or a combination of both for 5 minutes. Phospho-Stat1 tyrosine 701, phospho-Stat1 serine 727 and phospho-p38 MAPK threonine 180/tyrosine 182 were determined by western blot. Vinculin was used as loading control. Similar results were observed in 3 independent experiments. KD denotes knocked down.

In IFN signaling, all IFNs induce Stat1 activation through receptor-bound Janus kinase (Jak)-mediated phosphorylation of tyrosine 701. The IFN-γ receptor employs Jak1 and Jak2 to phosphorylate Stat1 exclusively, causing its homodimerization and nuclear translocation to promote expression of interferon-stimulated genes [49]. As expected, five minutes treatment of IFN-γ but not TNF-α induced Jak1 and Jak2 tyrosine phosphorylation (Figure 6A). The Jak1/Jak2 dual knock down by siRNA inhibited IFN-γ-induced Stat1 tyrosine phosphorylation.

On the other hand, neither TNF-α-induced Stat1 serine phosphorylation, nor p38MAPK phosphorylation was affected. IFN-γ alone was not able to induce Stat1 serine phosphorylation or p38MAPK phosphorylation (Figure 6A). The C-terminal serine 727 is a known target for p38MAPK [50]. The involvement of p38MAPK in Stat1 phosphorylation was further demonstrated by the preincubation of the cells with the inhibitor BIRB796 [51]. As shown in Figure 6B, BIRB796 inhibited TNF-α-induced Stat1 serine phosphorylation but had no effect on IFN-γ-induced Stat1 tyrosine phosphorylation (Figure 6B).

### TNF-α and IFN-γ in combination with HER2/HER3 blockade upregulates Th1 chemokine, CXCL-10 in a time-dependent manner

**Figure 7:**
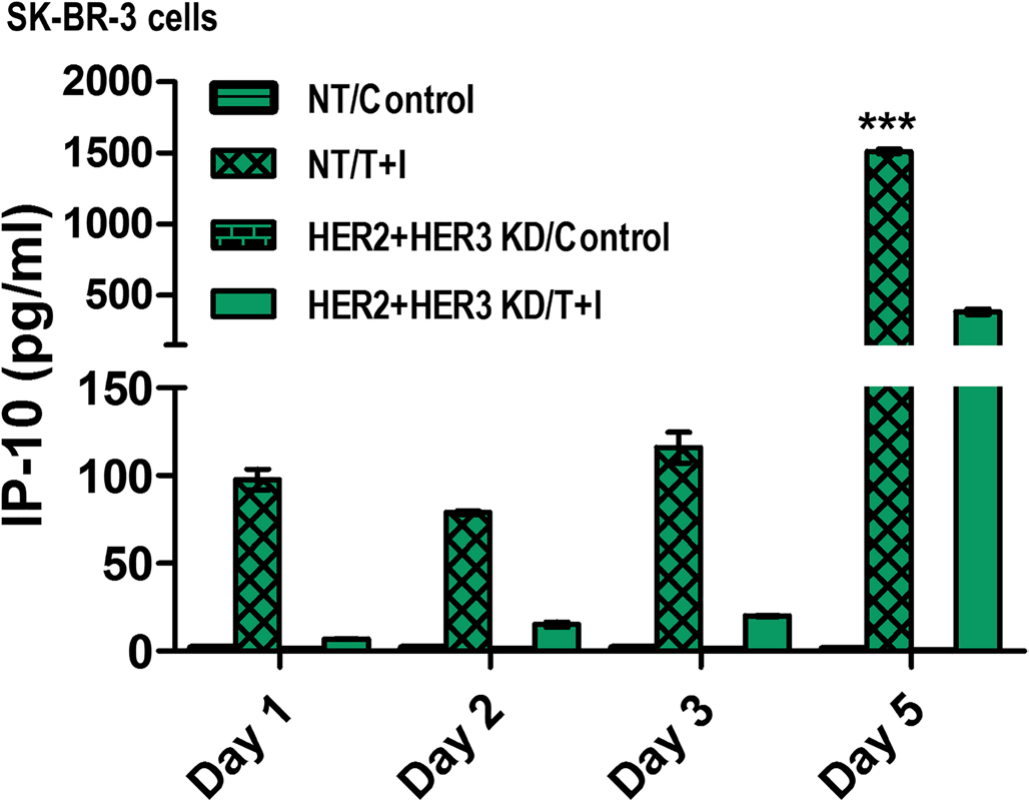
TNF-α and IFN-γ in combination with HER2/HER3 blockade upregulates Th1 chemokine, IP-10 in a time-dependent manner SK-BR-3 cells transfected with non-target (NT) or HER2/HER3 siRNA and were untreated or treated with 10 ng/ml TNF-α and 100 U/ml IFN-γ (T+I). Conditioned culture supernatants were collected at the indicated days after treatment for chemokine secretion measurement using an antibody array. Bar graphs represent mean ± SD (*n* = 3), ^***^p < 0.001. KD denotes knocked down.

We examined the effect of Th1 cytokines in combination with HER2/HER3 blockade on inducing chemokine secretion by senescence tumor cells. As shown in Figure 7, we observed time dependent secretion of Interferon gamma-induced protein 10 (CXCL10) by SK-BR-3 cells treated with Th1 cytokines TNF-α and IFN-γ compared to untreated cells. We did not observe detectable secretion of CXCL-10 in HER2/HER3 knock down cells. However, treatment of HER2/HER3 knock down cells with Th1 cytokines TNF-α and IFN-γ for five days significantly increased Th1 chemokine, CXCL-10 (Figure 7, *p* < 0.001). This data suggests that effect of Th1 cytokines not only induced tumor senescence but also induced CXCL-10 chemokine secretion which is known to be a chemoattractant for activated T cells that play an important dual role in recruiting activated T cells into sites of tissue inflammation and inhibiting angiogenesis [52, 53].

### IFN-γ and HER2/HER3 blockade results in senescence and apoptosis induction

**Figure 8:**
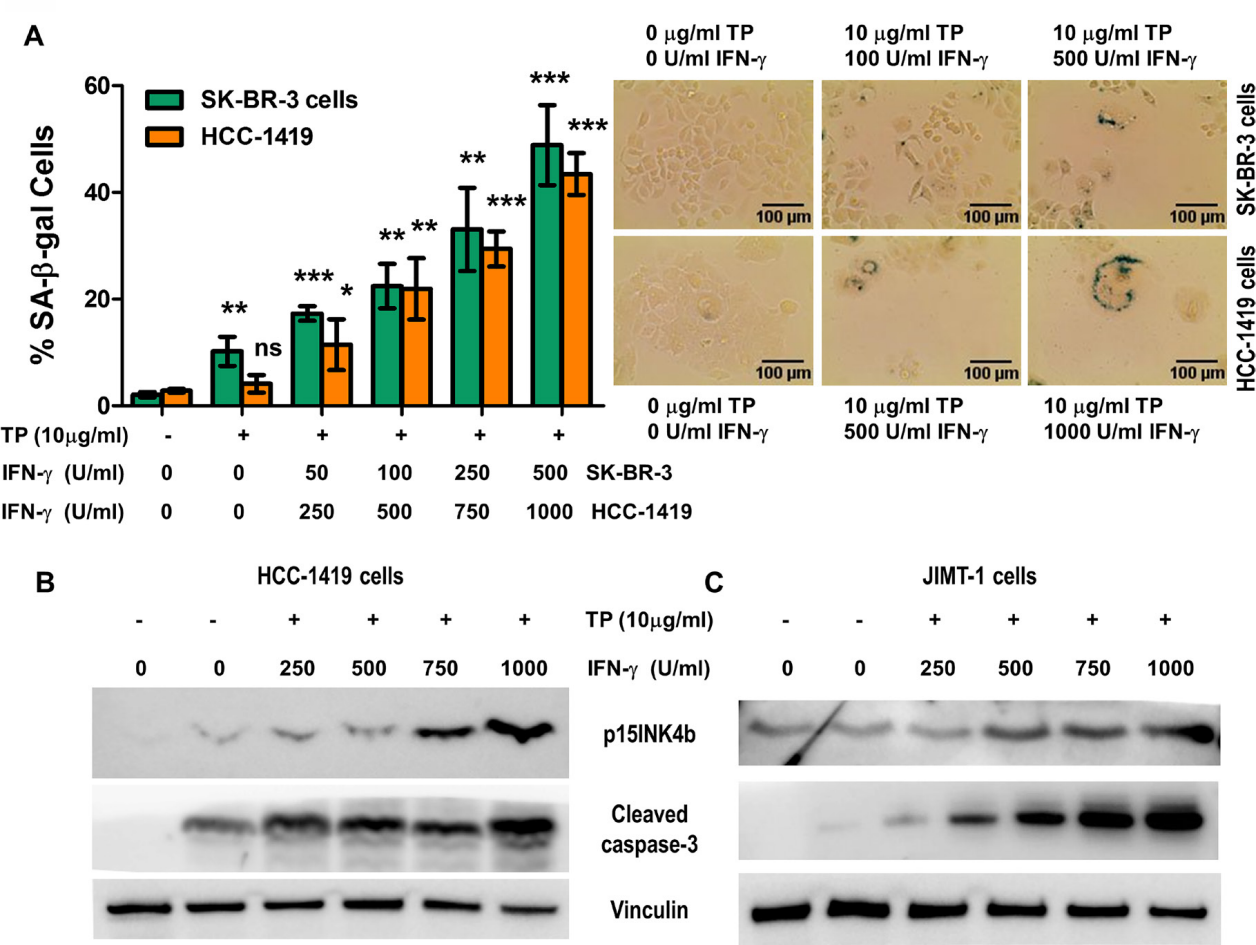
IFN-γ with trastuzumab and pertuzumab induce senescence and apoptosis in breast cancer cells (**A**) SK-BR-3 and HCC-1419 cells, untreated, or treated with 10 ug/ml of trastuzumab and pertuzumab (TP), and incubated with increasing concentrations IFN-γ for SK-BR-3 and HCC-1419. *Left panel*, densitometric analysis presented as % of SA-β-gal-positive cells, mean ± SD (*n* = 3), ^*^p < 0.05, ^**^p < 0.01, ^***^p < 0.001; ns, not significant. *Right panel*: representative data from 1 of 3 independent experiments in SK-BR-3 cells (*top panel*) and HCC-1419 cells (*bottom panel*). (**B** and **C**) p15INKb and cleaved caspase-3 expression of SK-BR-3 (B) and HCC-1419 (C) cells described in A. Vinculin was used as loading control. Similar results were observed in 3 independent experiments.

Taking into account that the systemic clinical use of TNF-α is impractical due to toxicity [54–56] and that IFN-γ is widely used clinically [57–59], we studied treatment of breast cancer cells with trastuzumab and pertuzumab and IFN-γ. Although the treatment with IFN-γ alone did not induce significant senescence or apoptosis (Figure 1, Supplementary Figure 1 and data not shown), we found that adding IFN-γ to trastuzumab and pertuzumab increased SA-β-gal staining (Figure 8A), p15INK4b expression and cleaved caspase-3 expression in SK-BR-3 (Figure 8B) and HCC-1419 (Figure 8C) cells.

### Th1 cytokines induce senescence and apoptosis in triple negative breast cancer cells

**Figure 9:**
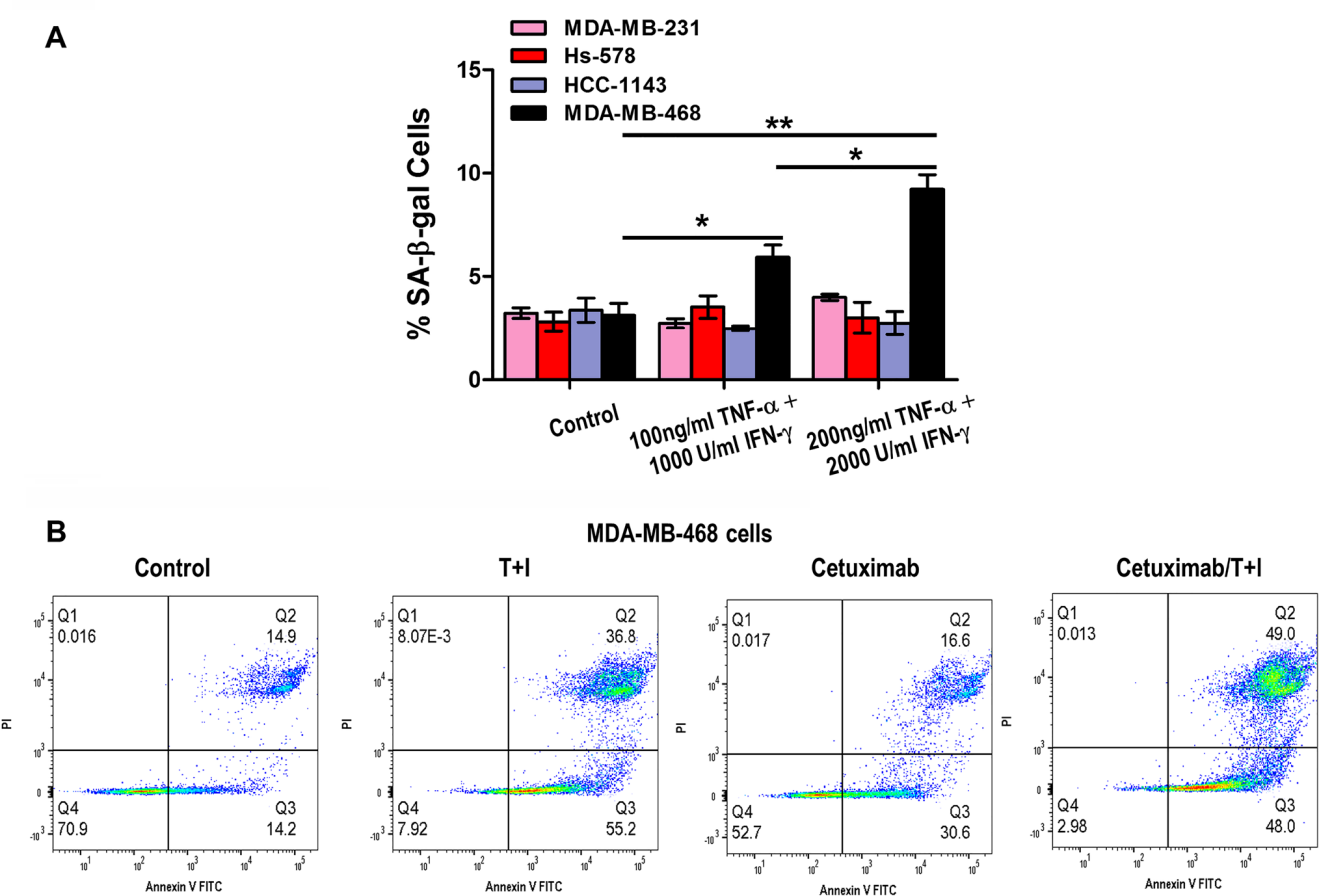
Th1 cytokine treatment induces apoptosis and senescence in triple negative breast cancer cells (**A**) MDA-MB-231, Hs-578, HCC-1143 and MDA-MB-468 cells untreated or treated with increasing concentrations of TNF-α and IFN-γ. Densitometric analysis presented as % of SA-β-gal-positive cells, mean ± SD (*n* = 3), ^*^p < 0.05, ^**^p < 0.01. Representative data from 1 of 3 independent experiments are presented. (**B**) MDA-MB-468 cells were treated with 200 ng/ml TNF-α (T) and 2000 U/ml IFN-γ (Ι), alone or in combination with 200 μg/ml cetuximab. Induction of apoptosis was visualized by staining for annexin V and PI expression of cells, followed by flow cytometry analysis and analyzed by FlowJo^®^ software. Plots are representative data from 1 of 3 independent experiments.

The group of tumors that do not express ER, PR, and do not have HER-2/Neu amplification is referred to as triple-negative breast cancer (TNBC), based on the lack of these three molecular markers. In general, hormone receptor expressing breast cancers have a more favorable prognosis than either those with HER-2/Neu amplification or those that are triple-negative [60, 61]. We found that high Th1 cytokines concentrations induced minimal senescence or apoptosis in MDA-MB-231, Hs-578 and HCC-1143 TNBC that mimic a high cancer stage (Figure 9A). A fourth triple negative cell line MDA-MB-468 presented minimal response to high doses to TNF-α and IFN-γ to induce senescence (Figure 9A, *p* < 0.05 and *p* < 0.01).

Cetuximab is a chimeric IgG monoclonal antibody that binds to EGFR and inhibits EGFR phosphorylation and activation [62]. Cetuximab (Erbitux) has been tested for efficacy in TNBC in multiple pre-clinical and clinical trials, alone and in combination with cytotoxic drugs [62–64]. In a TNBC xenograft murine model, cetuximab increased antitumor effect of doxorubicin [65]. Addition of cetuximab to carboplatin/irinotecan chemotherapy increased response rate in patients in a randomized phase II clinical trial from 30 to 49% [66]. In another randomized phase II trial with metastatic TNBC patients, cetuximab addition to cisplatin chemotherapy doubled overall response rate and longer progression free survival in patients [67].

Based on our senescence assay observation, we performed annexin V/PI staining assay to detect apoptosis in MDA-MB-468 cells, treated with TNF-α and IFN-γ alone and in combination with EGFR antibody cetuximab. While treatment with cetuximab alone did not enhance percentage of apoptosis in total cell population, combination of cetuximab with TNF-α and IFN-γ resulted in a larger population of apoptotic cells, with respect to untreated and cetuximab alone treated cells (Figure 9B).

### Combined EGFR and HER3 blockade sensitizes Th1 cytokine resistant triple negative breast cancer cells to senescence and apoptosis induction

**Figure 10:**
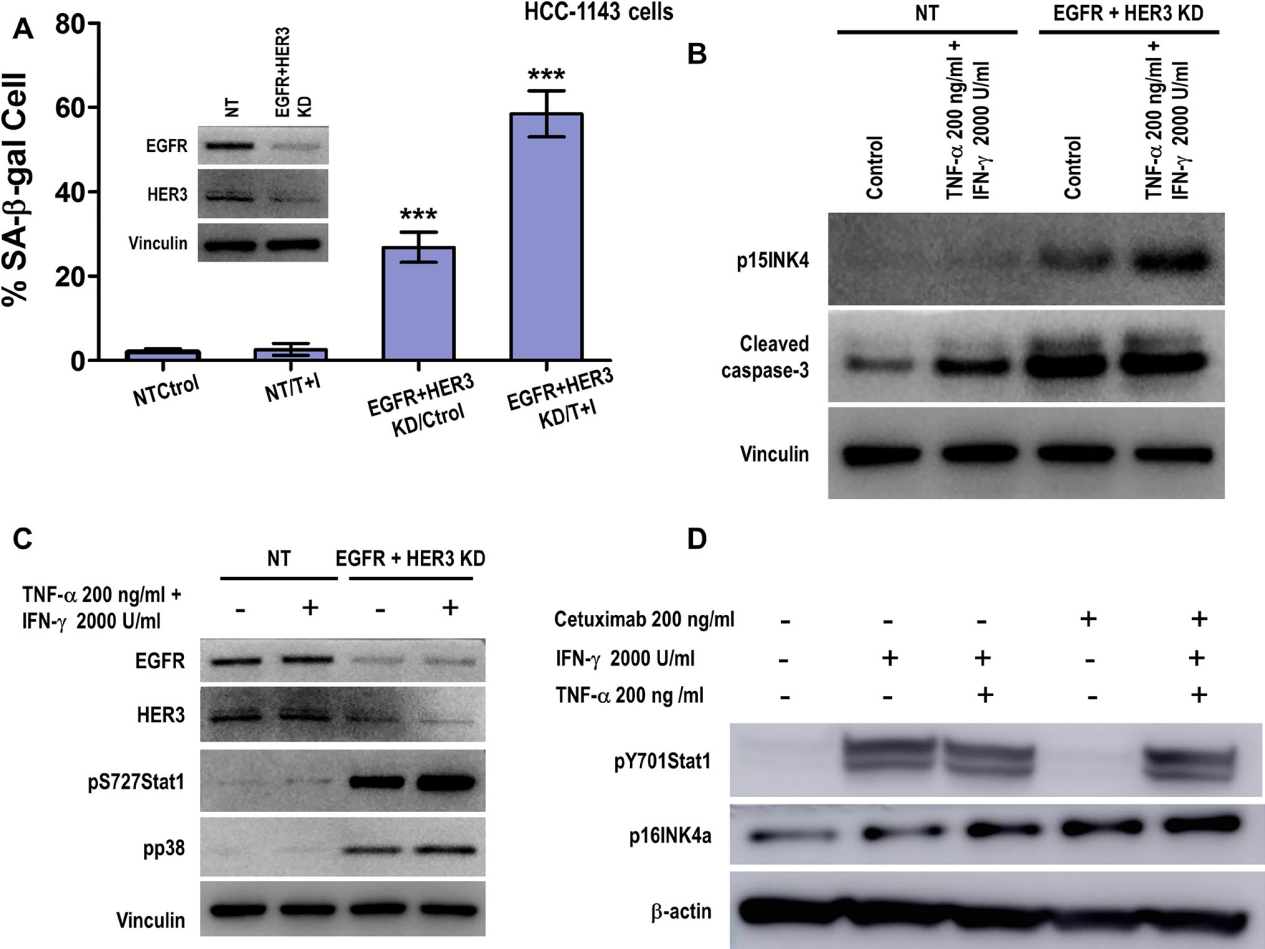
Combined EGFR and HER3 blockade sensitizes Th1 cytokine resistant triple negative breast cancer cells to senescence and apoptosis induction (**A**) Densitometric analysis presented as % of SA-β-gal-positive HCC-1143 cells transfected with non-target (NT) or EGFR and HER3 siRNA, untreated or treated with 200 ng/ml TNF-α (T) and 2000 U/ml IFN-γ (Ι), mean ± SD (*n* = 3), ^***^p < 0.001. *Inset:* HCC-1143 cells transfected with non-target (NT) or EGFR and HER3 siRNA probed with EGFR and HER3 specific antibodies. Vinculin was used as loading control. Representative data from 1 of 3 independent experiments. (**B**) Expression of p15INKb or cleaved caspase-3 in cells described in A. Vinculin was used as loading control. Similar results were observed in three independent experiments. (**C**) HCC-1143 cells were transfected with non-target (NT) or EGFR and HER3 siRNA, untreated or treated with 200 ng/ml TNF-α and 2000 U/ml IFN-γ for 5 min. EGFR and HER3 expression, phospho-Stat1 serine 727 and phospho-p38 MAPK threonine 180/tyrosine 182 were determined by western blot. Vinculin was used as loading control. Similar results were observed in three independent experiments. KD denotes knocked down. (**D**) Expression of phospho-Stat1 tyrosine 701 and p16INK4a proteins were detected in HCC-1143 cells treated with 200 ng/ml cetuximab for 48 hours followed by 200 μg/ml TNF-α and 2000 U/ml IFN-γ for 5 minutes. β-actin was used as loading control. Similar results were observed in 3 sets of independent experiments.

Taking into account that all these TNBC cells overexpress EGFR (Figure 1D), we reasoned that combining EGFR and HER3 blockade with high TNF-α (200 ng/ml) and IFN-γ (2000 U/ml) treatment would induce senescence and apoptosis similarly as HER2/HER3 inhibition combined with Th1 cytokines in HER2-expressing cell lines. As shown in Figure 10, EGFR/HER3 depletion in HCC-1143 cells induced senescence by β-gal staining (Figure 10A, *p* < 0.001), p15INK4b expression and apoptosis by caspase-3 activation (Figure 10B) compared to control and cytokine alone treated cells. Following the idea of the mechanism proposed in HER2-expressing breast cancer cells (Figure 5 and Supplementary Figure 6), we found that cells were resistant to the treatment with Th1 cytokines for five minutes but knocking down the oncodrivers EGFR and HER3 with siRNA induced Stat1 serine phosphorylation as well as p38MAPK, in concordance with the senescent and apoptotic phenotype (Figure 10C). In a separate experiment, HCC-1143 cells were treated with EGFR monoclonal antibody cetuximab for 48 hours to inhibit EGFR-mediated intracellular signaling, followed by five minutes of treatment with IFN-γ alone or TNF-α and IFN-γ. We observed IFN-γ treatment alone and in combination with TNF-α and cetuximab enhanced phosphorylation of Stat1 on tyrosine 701 and p16INK4a (Figure 10D). Cetuximab treatment alone did not increase Stat1 tyrosine phosphorylation, but enhanced p16INK4a expression in HCC-1143 TNBC cells, suggesting influence on cellular senescence in combination with Th1 cytokines. Similar results were observed in MDA-MB-468 TNBC cells (Supplementary Figure 6, *p* < 0.01, *p* < 0.001). We also detected Stat1 tyrosine phosphorylation by Th1 cytokine treatment alone or in combination with cetuximab in MDA-MB-231 cells (Supplementary Figure 7).

## DISCUSSION

Th1 cytokines TNF-α and IFN-γ are increasingly recognized as critical mediators of CD4^+^ T-cell-driven anti-tumor activity [68–72]. Rakhra *et al* proposed that the combined action of IFN-γ and TNFR1 signaling on endothelia-induced tumor dormancy may be mediated by antiangiogenic chemokines that arrest or delay tumor angiogenesis and subsequent multistage carcinogenesis. In absence of either IFN-γ or TNFR1 signaling, tumor-protective Th1 cells strongly enhanced multistage carcinogenesis [73]. Our results suggest that TNF-α and IFN-γ induce senescence and apoptosis which may provide a mechanism by which these cytokines inhibit multistage carcinogenesis.

We demonstrate that TNF-α and IFN-γ induce senescence and apoptosis in HER2-expressing breast cancer cells in a dose-dependent manner. Cytokine receptors were expressed at similar levels in all of the breast cell lines that were tested, implying that the variable susceptibility to cytokine-mediated senescence is not explained by variable expression of the receptors. These findings were not limited to high HER2 expressing cells but also intermediate HER2 ER^pos^ cells like MCF-7 and T-47D. In addition, the effect of Th1 cytokines could also be noted in TNBC (HER2^low^) cells, where EGFR was the expressed oncodriver. Th1 cytokines alone had minimal effect on senescence and apoptosis. Increased cell death and senescence was detected when Th1 cytokines (TNF-α and IFN-γ) were combined with EGFR antibody cetuximab (Figure 9B), suggesting a putative oncogene addiction in TNBC cells for EGFR, that caused apoptosis increased by combined oncodriver blockade and Th1 cytokines treatment.

In HER2^high^ or HER2^intermediate^ cells, silencing the HER2 gene increased senescence and apoptosis via oncogene inactivation and similarly inhibiting EGFR in triple negative cells (HER2^low^). Oncogene addiction, which describes the dependency of some cancer cells on a single activated oncogenic protein or pathway for the maintenance of the malignant phenotype [24, 74, 75], may leave tumors vulnerable in absence of the oncogene. For example, in hematopoietic tumors, osteosarcomas and hepatocellular carcinomas, inactivation of the oncogene MYC induced cellular senescence as an important mechanism of sustained tumor regression [76]. Oncogene inactivation has been proven to induce apoptosis in tumors by increasing pro-apoptotic pathways [75].

Thus, two pathways appear to facilitate senescence and apoptosis: stimulation of the immune system by Th1 cytokines and inhibition of the oncodrivers. *In vivo*, these two processes may be linked - oncogene inactivation may lead to some senescence and apoptosis, but Th1 cytokines facilitate further augment these processes and also induce Th1 chemokine production to further drive the immune response against senescent cells. This process has also been exemplified in murine models; MYC inactivation alone in immune-deficient mice was not able to induce cellular senescence without the presence of CD4^+^ T-cells [77]. Similarly, T-cell cytokines, TNF-α and IFN-γ, synergized with oncogene inhibition with a BRAF^V600E^ inhibitor, vemurafenib, to induce cell cycle arrest in melanoma [78]. We have recently showed that Th1 cytokines and trastuzumab may increase apoptosis by synergistically upregulating MHC class I on HER2^low^ cells [79]. Our findings suggest that EGFR inhibition with cetuximab for 48 hours followed by Th1 cytokine treatment induce senescence, as indicated by increased p16INK4a protein expression. This effect on p15INK4b and p16INK4a expression and cellular senescence was maintained even after five days of treatment and after multiple passage of culturing cells without Th1 cytokines. This effect also altered the expression levels of intracellular signaling mediator proteins (increased Stat1 phosphorylation and cleaved caspase-3, decreased Stat3 phosphorylation), suggesting early activation of apoptosis and anti-proliferative signaling pathways. Taken together, our data suggests combination of oncodriver (EGFR/HER3 or HER2/HER3) blockade with Th1 cytokine treatment induces an early and pronounced response of apoptosis and senescence.

The HER2 oncogene has been shown to collaborate with other members of the HER family, and multi-targeted blockade of the HER-family has been shown to increase anti-tumor activity. Nahta *et al* demonstrated that the combination of monoclonal antibodies trastuzumab and pertuzumab synergistically inhibits survival of BT-474 breast cancer cells by increasing apoptosis and inhibiting cell proliferation [42]. *In vivo*, the combination strongly enhanced antitumor effect and induced tumor regression in two xenograft models of breast and lung cancer [43]. Clinically, the use of both antibodies together constituted the first combination to be FDA-approved; they could be administered in the same dosages in combination as in monotherapy with limited overlapping toxicity and few pharmacokinetic interactions [44, 80–82].

The senescence and apoptosis induced by IFN-γ treatment in Figure 8 was not as great as when the cells were treated with both TNF-α and IFN-γ; however, treatment with IFN-γ alone is feasible to test in clinical applications in combination with current HER2 targeted agents.

**Figure 11:**
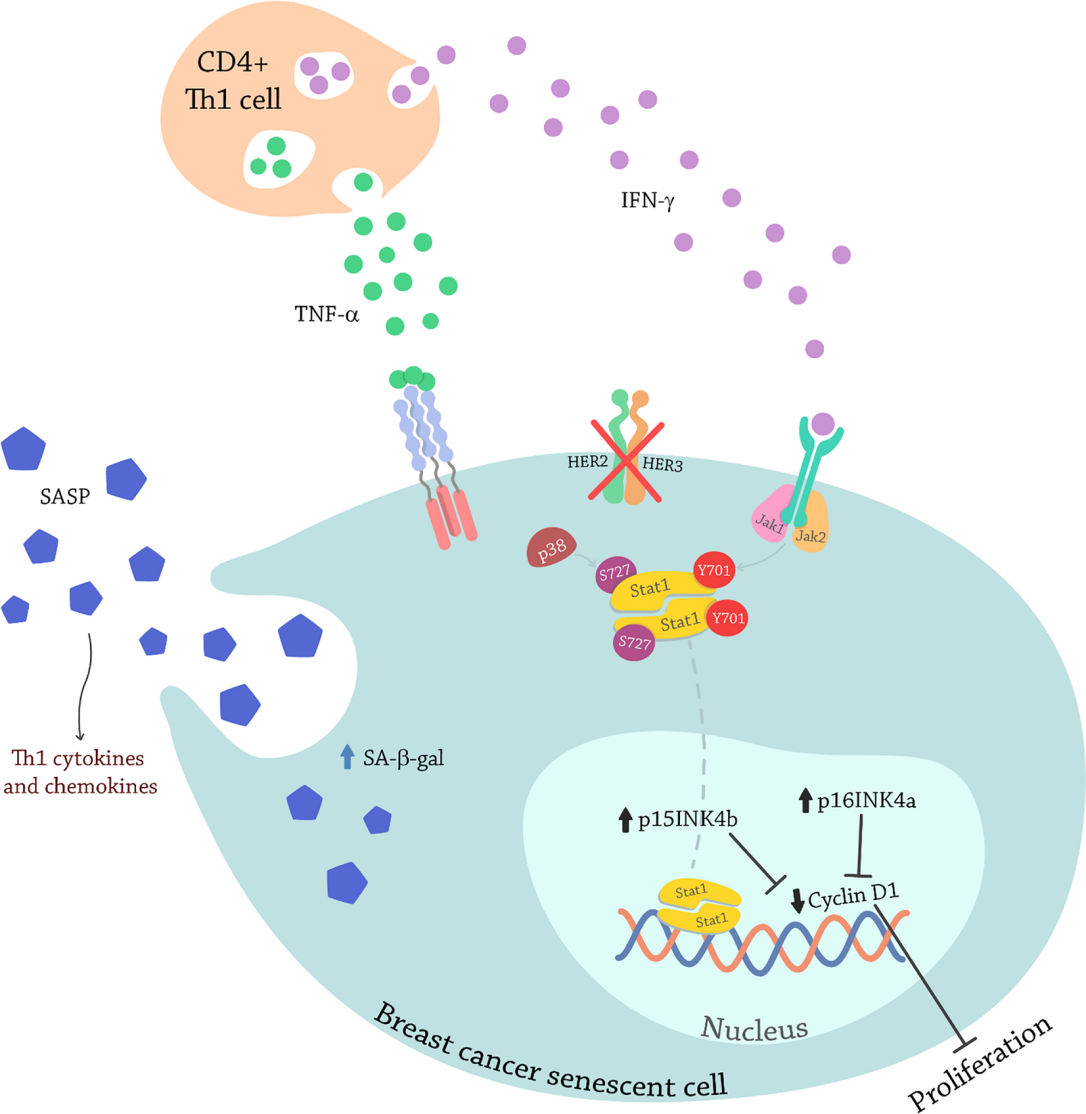
Model illustrating senescence induction by Th1 cytokines TNF-α and IFN-γ through Stat1 activation in breast cancer cells C4^+^ T-cells secret IFN-γ, that interacts with its receptors on the cancer cell inducing the tyrosine phosphorylation of Jak1 and Jak2. The Jaks now activated, induce the phosphorylation of Stat1 on tyrosine 701. At the same time, the C4^+^ T-cells are secreting TNF-α that also interacts with it receptors on the cancer cells, that induces the phosphorylation of p38MAPK. Blocking oncodrivers HER2/HER3 also promotes p38MAPK phosphorylation that induces the phosphorylation of Stat1 on serine 727. Stat1 dimerizes and fully activated on both residues translocates to the nucleus were acts as a transcription factor to promote senescence gens transcription. The senescent cell secretes proinflammatory cytokines and chemokines that attract more immune cells.

Our study provides evidence illustrated in Figure 11. IFN-γ, secreted by CD4^+^ T-cells, interacts with its receptors on the cancer cell surface and induces tyrosine phosphorylation of Jak1 and Jak2. Once the Jaks are activated they induce phosphorylation of Stat1 in tyrosine 701. Simultaneously TNF-α, also secreted by CD4^+^ T-cells, interacts with its receptors as well as inducing phosphorylation of p38MAPK. TNF-α has been shown to act synergistically with five new anti-HER2 antibodies to inhibit the growth of SK-BR-3 cells [83]. Additionally, inhibition of HER2/HER3 promotes activation of p38 and the combination with TNF-α and IFN-γ induces a higher p38MAPK phosphorylation. This event phosphorylates Stat1 on serine 727. Activated Stat1 dimerizes, translocates to the nucleus and fully activated by phosphorylation on both residues, binds to DNA to induce the transcription of senescent markers as p15 and p16 that are cell cycle inhibitors. This subsequently results in inhibition of transcription of cell cycle enzymes, such as Cyclin D1, inhibiting proliferation [84]. At the same time, there is an increase in SA-β-gal expression and the cells develop blue stain suggesting senescence induction. Finally, the Th1 cytokines cause early senescent cell to secrete Th1 chemokines that further attract immune cells (Figure 7) [29–32, 85].

We have recently showed that multivalent targeting of HER family Th1 cytokines may be necessary for CD8^+^ T-cell mediated cytotoxicity, although we did not specifically study senescence [79]. Here we showed that multi-targeted oncogene inhibition further enhanced senescence and apoptosis induced by CD4^+^ Th1 cytokines, TNF-α and IFN-γ. Combined HER2 and HER3 siRNA transfected breast cancer cells or combined trastuzumab and pertuzumab treated breast cancer cells demonstrated enhanced Th1 cytokine-induced senescence and apoptosis. Furthermore, treatment of trastuzumab and pertuzumab-resistant breast cancer cells with TNF-α and IFN-γ restored sensitivity and induced senescence and apoptosis. Fan *et al* have suggested that IFN-γ may restore sensitivity by upregulating HER2/neu expression, increasing the binding of ^131^I-Herceptin, and improving the inhibitory effect of ^131^I-Herceptin on proliferation of breast cancer cells [86]. Also, we found that combining EGFR/HER3 siRNA treatment in TNBC cells restore CD4^+^ Th1 cytokines sensitivity to senescence and apoptosis.

On the other hand, TNBC cells showed sensitivity towards IFN-γ treatment alone and in combination with TNF-α and cetuximab. Therefore, EGFR blockade with Th1 cytokines may offer a treatment approach in TNBC, where tumor cells grow rapidly and recurrence is significantly faster than other sub-types of breast cancers. Anti-EGFR agents have been used with mixed results in chemotherapy [62, 66, 67, 87, 88]. Our data suggests critical cooperativity between oncodriver blockade and Th1 cytokines in causing senescence of tumor cells. The recent identification of the class of senolytic drugs [89, 90] suggests further combinations may have therapeutic potential.

We have recently demonstrated a progressive loss of the anti-HER2 CD4^+^ Th1 immune response along the continuum of HER2 positive breast tumorigenesis; depressed Th1 responses correlate with worse clinico-pathological outcomes [20]. Although this immune deficit is not affected by surgery, radiation, or chemotherapy, it can be restored by type 1-polarized dendritic cell (DC1) vaccination. In the present study, we showed that HER2 specific CD4^+^ Th1-cells, when encountering antigen, induce senescence and apoptosis in HER2-expressing breast cancer cells. Moreover, the effect was significantly enhanced with the addition of trastuzumab and pertuzumab. These results support rational combinations of Th1-directed immune interventions (e.g., DC1 vaccine approaches [45]) with approved breast cancer treatments [91]. Alternative replacement of anti-HER2 CD4^+^ Th1 would be to administer Th1 cytokines such as systemic IFN-γ or IFN-γ-inducing agents like STING and Toll like receptor agonists, may be combined with anti-HER2 targeted therapies as an immunotherapeutic approach to treat HER2 positive cancer patients [92, 93]. Our results in triple negative cells demonstrated that this approach is not exclusive of HER2-expressing cells and could be extended to other cancers. Interestingly we have also noted a similar loss in anti-HER3 CD4^+^ Th1 in TNBC and ER^pos^ breast cancer suggesting similar loses of anti-oncodriver Th1 may be a common theme in several breast cancer subtypes and replacing Th1 cytokines may be a critical component in breast cancer therapy in general.

In summary, our results establish a critical role for Th1 cytokines, TNF-α and IFN-γ, inducing tumor senescence and apoptosis in breast cancer, and demonstrate a complementary effect with oncogene inactivation that could potentially be applicable to other types of cancers. An effective CD4^+^ Th1 response, or INF-γ alone combined with oncogene blockade can significantly drive tumor senescence and apoptosis and should be explored as a non-cross-reactive therapy to effectively eliminate residual cancer cells and prevent recurrence in HER2-expressing and triple negative breast cancer. Restoring the anti-HER2 Th1 cytokine response or replacing its absence using alternative strategies may be critical to eliminate microscopic tumor deposits that serve as a source of recurrence.

## MATERIALS AND METHODS

### Cell culture and treatments

Human breast cancer cell lines SK-BR-3, BT-474, MCF-7, T-47D, HCC-1419, MDA-MB-231 and MDA-MB-468 were obtained from the American Type Culture Collection (Manassas, VA) and grown in RPMI-1640 (Life technologies, Grand Island, NY) supplemented with 10% fetal bovine serum (FBS, Cellgro, Herndon, VA). Hs-578 and HCC-1143 were a kind gift from Dr. Hatem Soliman (Moffitt Cancer Center, Tampa, FL) and were grown in the same complete medium. JIMT-1 cells were a kind gift from Dr. Pravin Kaumaya (Ohio State University, Columbus, OH) and were grown in Dulbecco’s modified Eagle’s medium (DMEM) (Invitrogen, Walham, MA) supplemented with 10% FBS. All cells were grown at 37° C in a humidified 5% CO_2_ incubator.

Three hundred thousand breast cancer cells were treated for five days with the indicated concentrations (10–200 ng/ml) of human recombinant TNF-α (BD Biosciences, San Jose, CA) and (100–2000 U/ml) human recombinant IFN-γ (BD Biosciences) and then cultured for 2 more passages in absence of cytokines. Cells were subjected to senescence associated β-gal enzyme (SA-β-gal) detection or lysed and subjected to western blot analysis for p15INK4b and p16INK4a and cleaved caspase-3.

In the indicated cases, cells were treated with 10 ug/ml trastuzumab and pertuzumab (Herceptin and Perjeta, respectively, Genentech, San Francisco, CA), 200ug/ml cetuximab (Erbitux, Eli Lilly, Indianapolis, IN). When indicated, cells were incubated with BIRB 796 (Millipore, Billerica, MA). These treatments were combined with cytokines or with human recombinant heregulin (HRG, R&D Systems, Minneapolis, MN).

Fifty thousand cancer cells were plated in the lower chamber of a transwell system (BD Biosciences) with 5 × 10^5^ human CD4^+^ T-cells and 0.5 × 10^5^ mature (i.e. type 1 polarized) or immature human dendritic cells (DCs) in the upper chamber. DCs and CD4^+^ T-cells were obtained from select trial subjects [45]. DCs and iDCs were pulsed with Class II-derived HER2 or control irrelevant (BRAF and survivin) peptides (20 µg/ml). Co-cultures were incubated for 5 days at 37° C. Control wells contained CD4^+^ T-cells only. Fifty thousand cancer cells were also incubated in the presence of DC/CD4^+^ T-cell co-culture supernatants for 5 days at 37° C. In both approaches, cells were cultured for 2 additional passages in absence of cytokines and subjected to senescence studies (SA-β-gal activity at pH 6 and p15INK4b and p16INK4a by western blot) and apoptosis studies (cleaved caspase-3 by western blot). Antibodies were added to cells 60 minutes before incubation with the co-culture of DC and CD4^+^ T-cells to neutralize Th1-elaborated cytokines: polyclonal goat IgG anti-human TNF-α (0.06 µg/ml per 0.75 ng/ml TNF-α) and IFN-γ (0.3 µg/ml per 5 ng/ml IFN-γ), and goat IgG isotype as the corresponding negative control (all from R&D Systems).

### RNA interference (RNAi) transfections

Small interfering RNA (siRNA) SMART Pool: ON TARGET Plus EGFR siRNA, HER2 siRNA, HER3 siRNA, Jak1 siRNA, JAk2 siRNA, and SMART Pool: ON-TARGET Plus Non-Targeting Pool were purchased from Dharmacon-Thermo Scientific. The following target sequences were used: EGFR: CAAAGUGUGUAACGGAAUA, CCAUAAAUGCUACGAAUAU, GUAACAAGCUCACGCAGUU, CAGAGGAUGUUCAAUAACU; HER2: UGGAAGAGAUCACAGGUUA, GAGACCCGCUGAACAAUAC, GGAGGAAUGCCGAGUACUG, GCUCAUCGCUCACAACCAA; HER3: GCGAUGCUGAGAACCAAUA, AGAUUGUGCUCACGGGACA, GCAGUGGAUUCGAGAAGUG, UCGUCAUGUUGAACUAUA; Jak1: GAAAAUGAAUUGAGUCGAU, GAAAUCACCCACAUUGUAA, CGCAUGAGGUUCUACUUUA, GCACAGGGACAGUAUGAUU; JAK2: AAUAGGAGACUUCGGAUUA, GAAUUGUAACUGUCCAUAA, GAACUUAGCUCAUUAAAAG, GAAUUUAUGCGAAUGAUUG; Non-targeting: UGGUUUACAUGUCGACUAA, UGGUUUACAUGUUGUGUGA, UGGUUUACAUGUUUUCUGA, UGGUUUACAUGUUUUCCUA. Three hundred thousand cells were transfected with siRNA sequences (25 nM) using RNAi Max Lipofectamine (Life Technologies) in serum free medium, and after 1 hour the medium was supplemented with 10% FBS. Sixteen hours later, cells were subjected to 48 hours of serum starvation followed by various designated treatments and western blot to check expression levels.

### SA-β-gal activity at pH 6

Cells were washed twice in PBS, fixed in 3% formaldehyde, and washed again in PBS. The cells were incubated overnight at 37° C (without CO_2_) with freshly prepared SA-β-gal staining solution from Millipore as per the manufacturer’s instructions. The percentage of SA-β-gal-positive (blue) cells in each sample was determined using a bright-field microscope (Evos Core_XL_, Bothel, WA/40X/2048 × 1536, 3.2 μm/pixel; 3.1 MP COLOR/ Captured images: Color TIFF, PNG, JPG or BMP-2048 × 1536 pixels)

### Western blot analysis

Lysates were prepared from cell lines, lysed in a buffer containing 50 mM Tris (pH 7.4), 150 mM NaCl, 1 mM EDTA, 1 mM EGTA, 10% glycerol, 70% Tergitol, 0.1% SDS, 1 mM Mg_2_Cl and protease inhibitor cocktail Sigma-Aldrich (St. Louis, MO). Lysates were centrifuged at 12,000 × *g* for 15 minutes at 4° C. Proteins were solubilized in sample buffer (Life Technologies) and subjected to SDS-PAGE. Proteins were electroblotted onto PVDF membranes and were immunoblotted with the following antibodies: p15INK4b (K-18), p16INK4a (50.1), IFN-γRα (C-20), HER3 (C-17) all from Santa Cruz Biotechnology (Santa Cruz, CA); Vinculin (V9131) from Sigma-Aldrich; EGFR (D38B1), HER2 (29D8), HER3/ErbB3 (1B2), cleaved caspase-3 (Asp175), TNF-R1 (C25C1), phospho-Akt (Ser473), Phospho-Stat1 (Tyr701) (58D6), phospho-Stat1 (Ser727), phospho-Stat3 (Tyr705) (M9C6), phospho-Jak1 (Tyr1022/1023), phospho-Jak2 (Tyr1007/1008) (C80C3), Phospho-p38 MAPK (Thr180/Tyr182) (D3F9) and β-actin (13E5) from Cell Signaling Technologies (Danvers, MA). After washing, membranes were incubated with HRP-conjugated secondary antibody (Bio-Rad, Hercules, CA). Bands were visualized and quantified by using the enhanced chemiluminescence (ECL) western blot detection system and the Image Reader LAS-1000 Lite version 1.0 software (Fuji). Quantification of western blots was performed using ImageJ software (http://rsb.info.nih.gov/ij/).

### Flow cytometric analysis

SK-BR-3 cells were treated with TNF-α (10 ng/ml) and IFN-γ (100 U/ml), trastuzumab (10 µg/ml) and pertuzumab (10 µg/ml), or a combination of TNF-α, IFN-γ, trastuzumab and pertuzumab for 24 hours. After incubation, apoptosis induction was determined using FITC-annexin V apoptosis detection kit (BD Biosciences) according to the manufacturer’s instructions.

MDA-MB-468 cells were grown for 24 hours before treatment with a combination of TNF-α and IFN-γ, alone and in combination with cetuximab (200 μg/ml). After 48 hours of treatment; apoptosis in untreated vs. treated cells was determined by FITC-Annexin V apoptosis detection kit.

### Chemokine detection

SK-BR-3 cells were transfected with NT or HER2/HER3 siRNA as described above and then treated with Th1 cytokines, TNF-α (10 ng/ml) and IFN-γ (100 U/ml). Culture supernatants were collected at 24, 48, and 72 hours and on day 5 after treatment for chemokine secretion using Legend Plex immunoassay kit following manufacturer’s instructions.

### Statistical analysis

Unpaired Student’s *t*-test (two-tailed) analysis was performed using GraphPad Prism (GraphPad Software, La Jolla, CA, USA). A *p*-value of 0.05 or less was considered significant - ^*^p < 0.05, ^**^p < 0.01, ^***^p < 0.001.

## SUPPLEMENTARY MATERIALS FIGURES



## Abbreviations

TNF-α: tumor necrosis factor alpha
IFN-γ: interferon gamma
TP: trastuzumab and pertuzumab
TNBC: triple negative breast cancer
Stats: signal transducers and activators of transcription
Th1: T-helper type 1
SA-β-gal: senescence associated acidic β-galactosidase
DC: dendritic cell
siRNA: small interference ribonucleic acid
Jak: Janus kinase
IP-10: Interferon gamma-induced protein 10
KD: knocked down
