# Supplementary material for: Oncodriver inhibition and CD4+ Th1 cytokines cooperate through Stat1 activation to induce tumor senescence and apoptosis in HER2+ and triple negative breast cancer: implications for combining immune and targeted therapies
Source: Oncotarget. 2018 May 1;9(33):23058–77. doi: 10.18632/oncotarget.25208 (PMC5955413; doi:10.18632/oncotarget.25208)
Supplement: Supplementary file 1 [file oncotarget-09-23058-s001.pdf]

Oncodriver inhibition and CD4+ Th1 cytokines cooperate through Stat1 activation to induce tumor senescence and apoptosis in HER2+ and triple negative breast cancer: implications for combining immune and targeted therapies

SUPPLEMENTARY MATERIALS

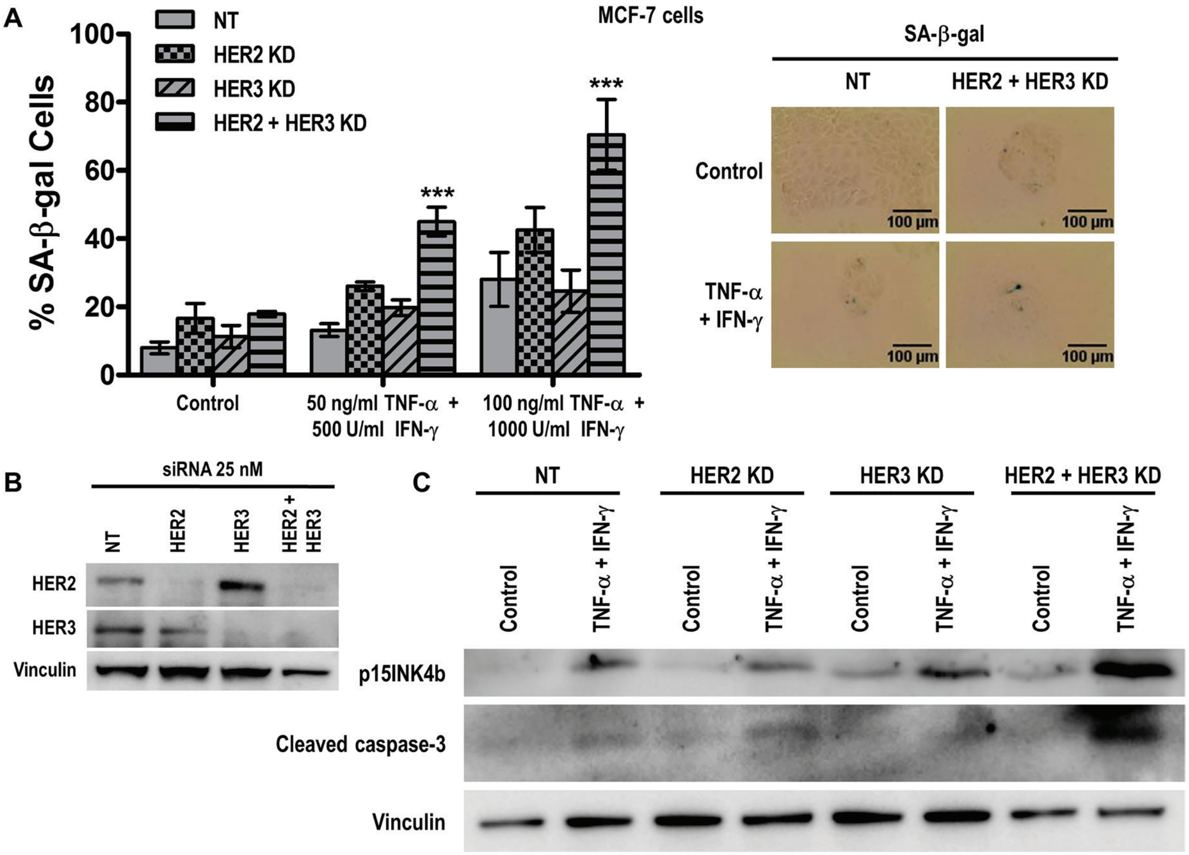

Supplementary Figure 1: Combined HER2 and HER3 blockade enhances Th1 cytokine-induced senescence and apoptosis in MCF-7 breast cancer cells. (**A**) MCF-7 cells transfected with non-target (NT), HER2, HER3, or both HER2 and HER3 siRNA, and then treated with TNF-α and IFN-γ. *Left panel:* densitometric analysis presented as % of SA-β-gal-positive cells, mean ± SD (n = 3), $^{***}p < 0.001$. *Right panel*: representative data from 1 of 3 independent experiments. (**B**) MCF-7 cells transfected with NT, HER2, HER3, or both HER2 and HER3 siRNA probed with HER2 and HER3 specific antibodies. (**C**) Expressions of p15INKb and cleaved caspase-3 proteins in cells described in A were analyzed by western blot. Vinculin was used as loading control. Similar results were observed in 3 independent experiments. KD denotes knocked down.

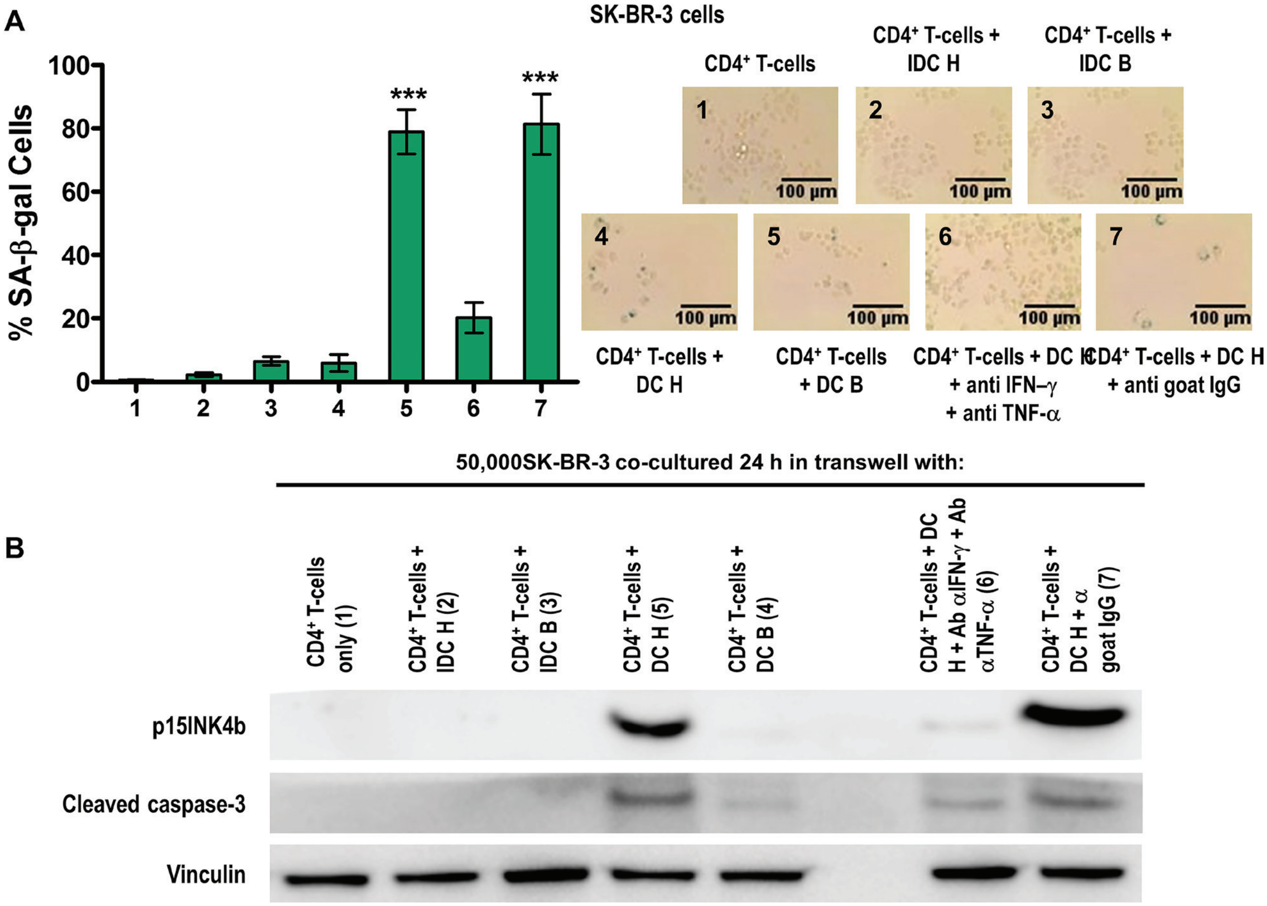

Supplementary Figure 2: Effect of Th1 cytokines on senescence and apoptosis. (**A**) SK-BR-3 cells were co-cultured with $CD4^+$ T-cells alone ($CD4^+$ only, 1), $CD4^+$ T-cells + mature type 1 dendritic cells (DC, 4-7) or immature DC (IDC, 2-3) pulsed with HER2 peptide (H, 2, 5, 6, 7) or irrelevant peptide (BRAF, B, 3, 4). Compared with an IgG isotype control, senescence induced in SK-BR-3 treated with $CD4^+$/DC H is partially rescued by neutralizing TNF-α and IFN-γ with specific antibodies (75.27 % rescue). *Left panel:* densitometric analysis presented as % of SA-β-gal-positive cells, mean ± SD (n = 3), $^{***}p < 0.001$. *Right panel:* Representative data from 1 of 3 independent experiments. (**B**) Increased p15INK4b and cleaved caspase-3 expressions suggest induced senescence and apoptosis, respectively, when co-cultured with DC H/$CD4^+$ T-cells, but not DC B, IDC H, and IDC B groups. Compared with IgG isotype control, senescence and apoptosis induced in SK-BR-3 treated with $CD4^+$/DC H were partially rescued by neutralizing TNF-α and IFN-γ with specific antibodies. Vinculin was used as loading control. Results are representative of 3 experiments.

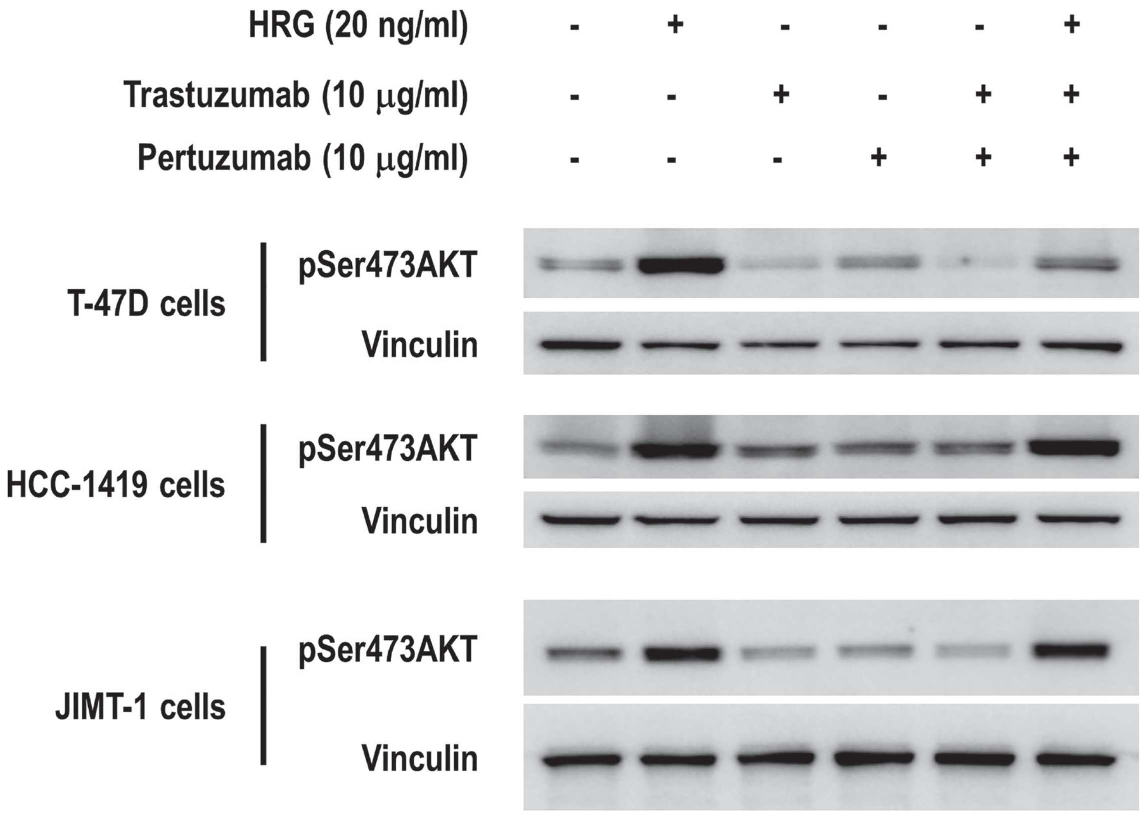

Supplementary Figure 3: Effect of trastuzumab and pertuzumab on AKT activation by heregulin in breast cancer cell lines. Serum-starved T-47D, HCC-1419 and JIMT-1 cells were treated with trastuzumab and pertuzumab for one hour and stimulated with HRG (five minutes). Cell extracts were subjected to western blot analysis using specific anti-phospho-AKT serine 473 antibody. Vinculin was used as loading control. Similar results were observed in 3 independent experiments.

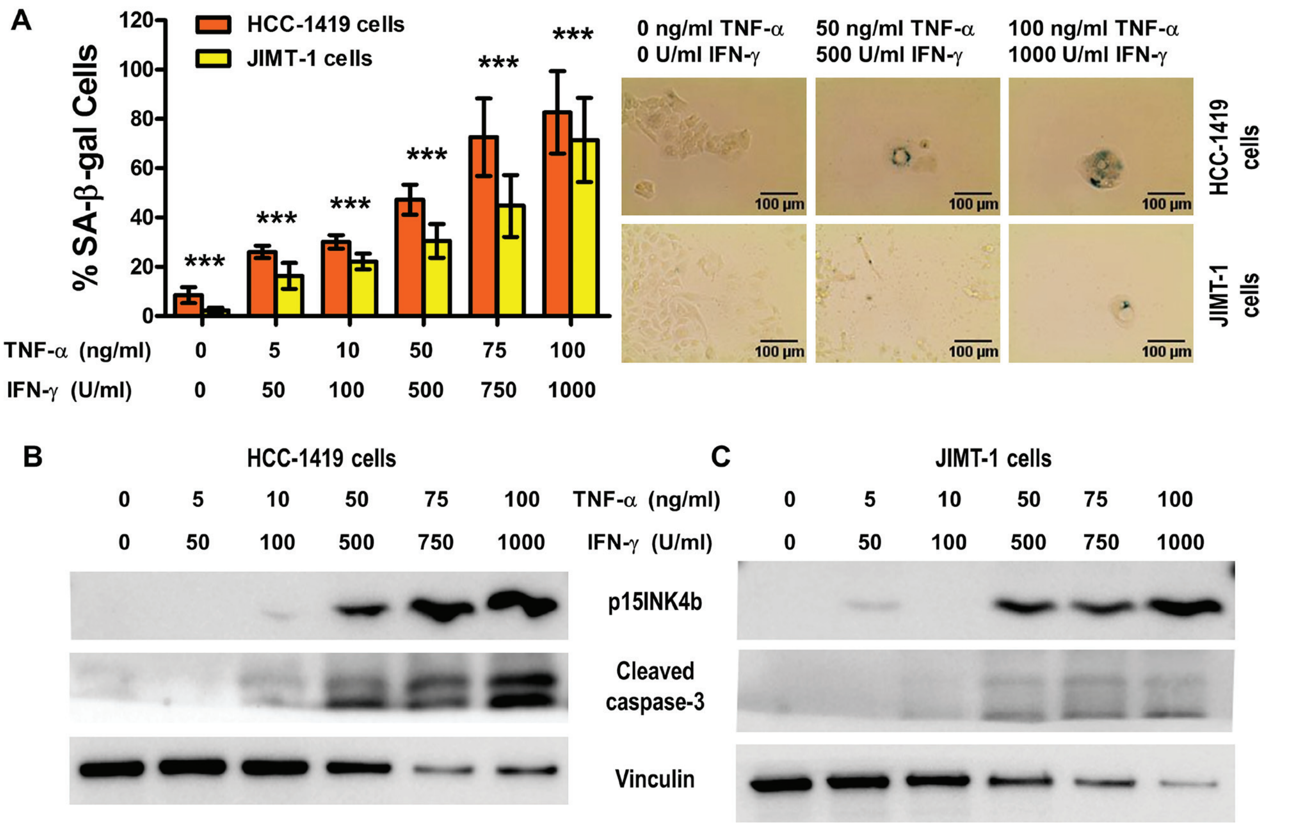

Supplementary Figure 4: Th1 cytokines TNF-a and IFN-g synergize to induce senescence and apoptosis in trastuzumab and pertuzumab resistant breast cancer cells. (**A**) HCC-1419 and JIMT-1 cells were untreated, or incubated with increasing concentrations of TNF-α and IFN-γ: 5 ng/ml and 50 U/ml, 10 ng/ml and 100 U/ml, 50 ng/ml and 500 U/ml, 75 ng/ml and 750 U/ml, 100 ng/ml and 1000 U/ml. *Left panel*, densitometric analysis presented as % of SA-β-gal-positive cells, mean ± SD ($n = 3$), $^{***}p < 0.001$. *Right panel*: representative data from 1 of 3 independent experiments in HCC-1419 cells (*top panel*) and JIMT-1 cells (*bottom panel*). (**B** and **C**) p15INKb and cleaved caspase-3 expression of HCC-1419 (B) and JIMT-1 (C) cells described in A. Vinculin was used as loading control. Similar results were observed in 3 independent experiments.

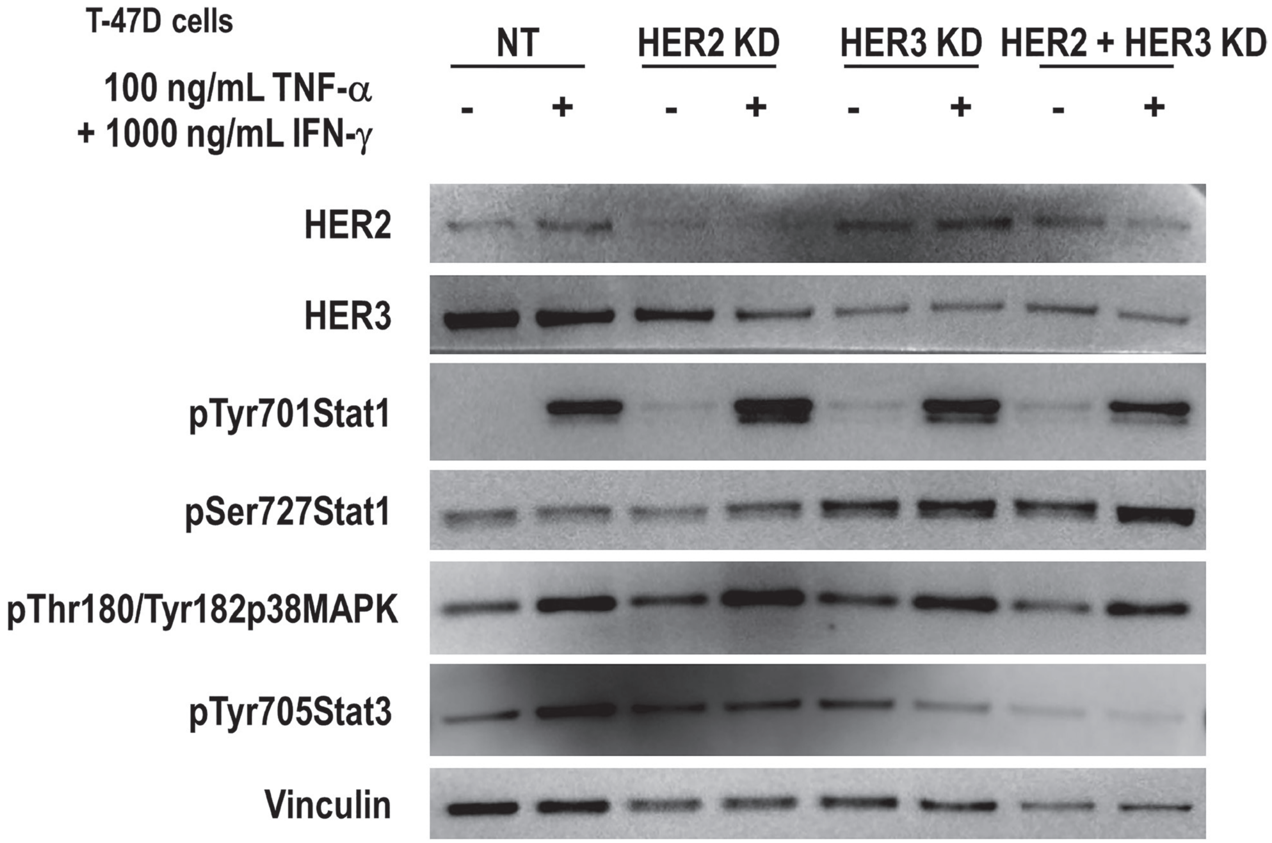

Supplementary Figure 5: Th1-mediated Stat1 activation and HER2 and HER3 blockade enhances Th1-mediated Stat1 activation. T-47D cells were transfected with non-target (NT), HER2, HER3 or HER2/HER3 siRNA, the cells were serum starved for 48 hours and untreated or treated with 100 ng/ml TNF-α and 1000 U/ml IFN-γ for 5 min. HER2 and HER3 expression, phospho-Stat1 tyrosine 701, phospho-Stat1 serine 727, phospho-Stat3 tyr 705 and phospho-p38 MAPK threonine 180/ tyrosine 182 were determined by western blot. Vinculin was used as loading control. Similar results were observed in 3 independent experiments. KD denotes knocked down.

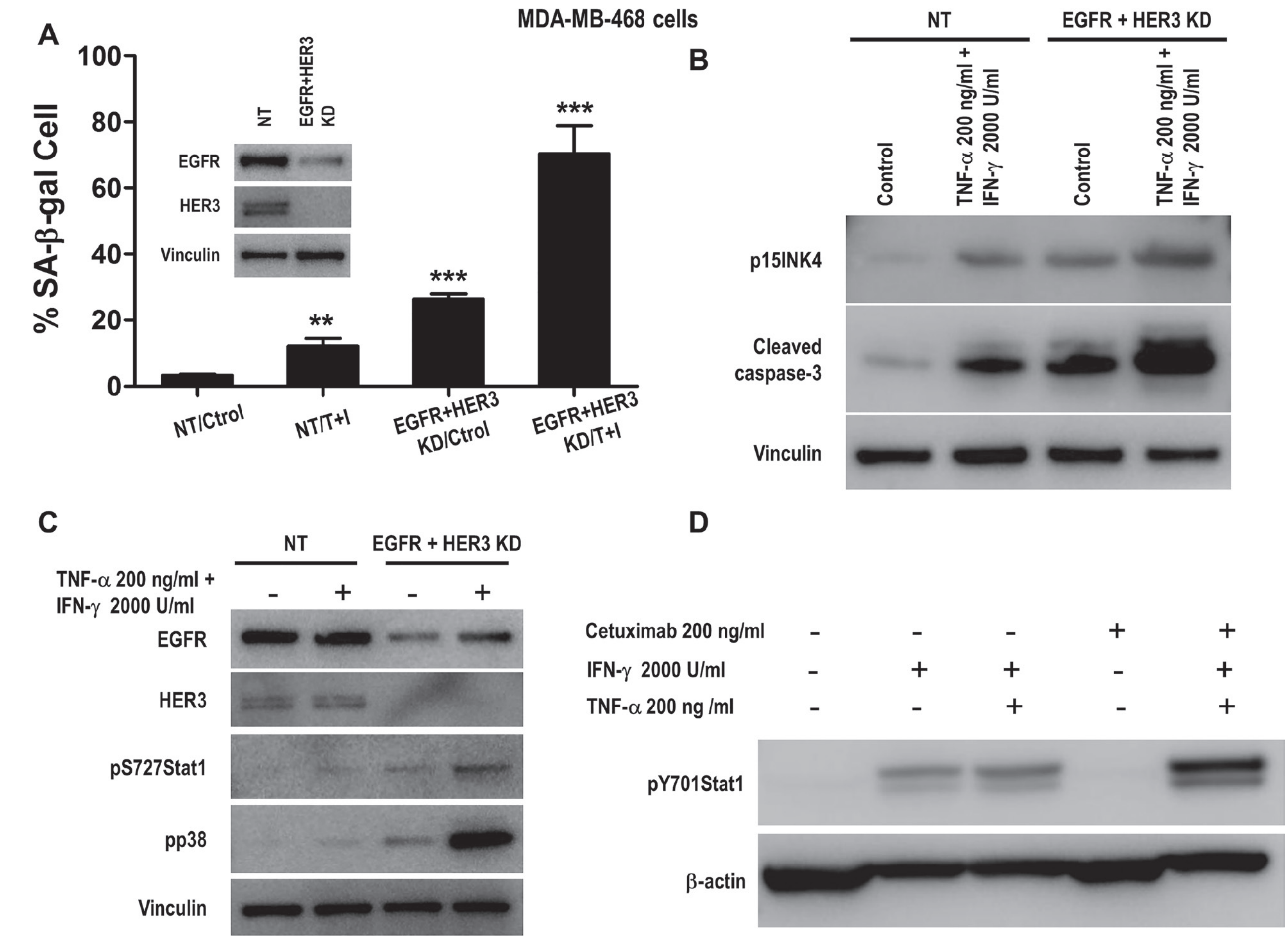

Supplementary Figure 6: Combined EGFR and HER3 blockade sensitizes Th1 cytokine resistant triple negative breast cancer cells to senescence and apoptosis induction. (**A**) Densitometric analysis presented as % of SA-β-gal-positive MDA-MB-468 cells transfected with non-target (NT) or EGFR and HER3 siRNA, untreated or treated with 200 ng/ml TNF-α (T) and 2000 U/ml IFN-γ (I), mean ± SD ($n = 3$), $^{**}p < 0.01$, $^{***}p < 0.001$. *Inset: M*DA-MB-468 cells transfected with non-target (NT) or EGFR and HER3 siRNA probed with EGFR and HER3 specific antibodies. Vinculin was used as loading control. (**B**) p15INKb or cleaved caspase-3 expression of cells described in A. Vinculin was used as loading control. Similar results were observed in 3 independent experiments. (**C**) MDA-MB-468 cells were transfected with non-target (NT) or EGFR and HER3 siRNA, untreated or treated with 200 ng/ml TNF-α and 2000 U/ml IFN-γ for 5 min. EGFR and HER3 expression, phospho-Stat1 serine 727 and phospho-p38 MAPK threonine 180/ tyrosine 182 were determined by western blot. Vinculin was used as loading control. Similar results were observed in 3 independent experiments. (**D**) Expression of phospho-Stat1 tyrosine 701 was detected in MDA-MB-468 cells treated with 200 ng/ml cetuximab for 48 hours followed by 200 ng/ml TNF-α and 2000 U/ml IFN-γ for 5 minutes. β-actin was used as loading control. Similar results were observed in 3 sets of independent experiments. KD denotes knocked down.

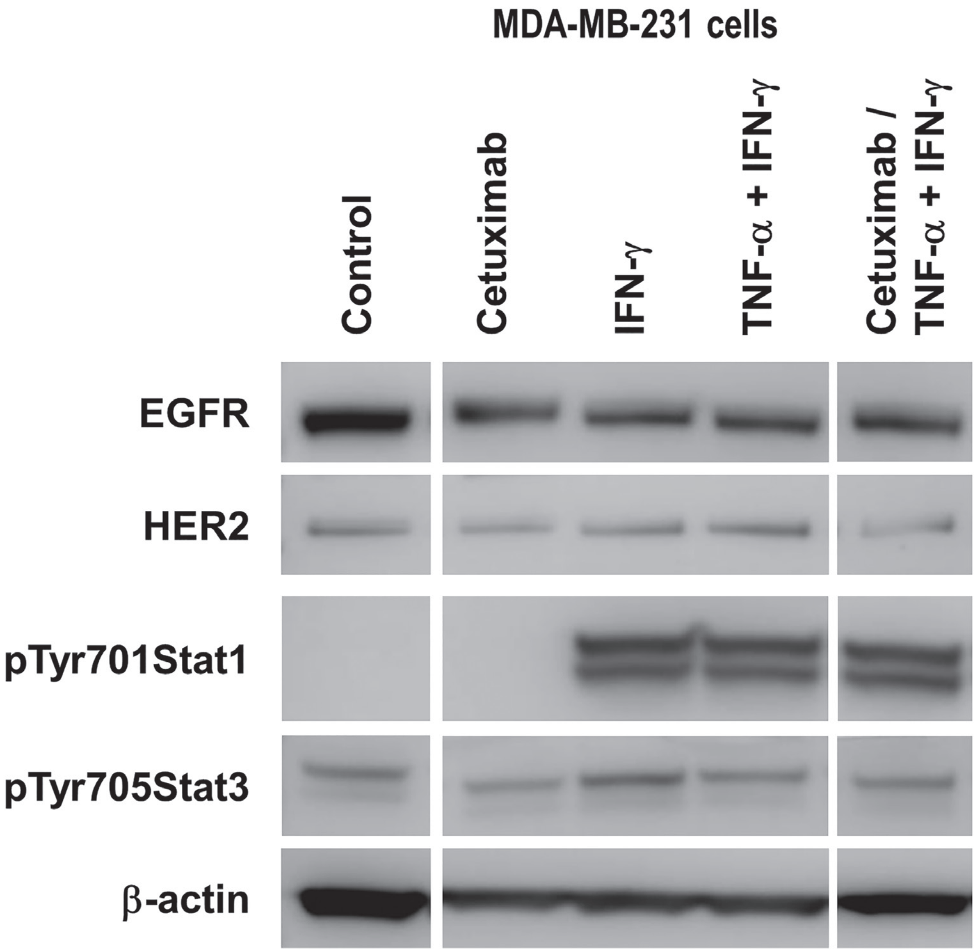

Supplementary Figure 7: TNBC cells are sensitive to Th1 cytokine treatment. MDA-MB-231 cells were treated with 200 ng/ml cetuximab for 48 hours followed by 200 ng/ml TNF-α and 2000 U/ml IFN-γ for 5 minutes. Expression of EGFR, HER2, phospho-Stat1 tyrosine 701 and phospho-Stat3 tyrosine 705 were determined by Western blotting. β-actin was used as loading control. Similar results were observed in 3 sets of independent experiments.
